# Multi-Residue Determination and Risk Assessment of EU-Relevant Pharmaceuticals, Pesticides, and UV-Filters in Drinking Water

**DOI:** 10.3390/ph19030402

**Published:** 2026-02-28

**Authors:** Inês M. Quintela, Ana M. Gorito, Marta O. Barbosa, Adrián M. T. Silva, Ana R. L. Ribeiro

**Affiliations:** LSRE-LCM, ALiCE, Faculty of Engineering, University of Porto, Rua Dr. Roberto Frias, 4200-465 Porto, Portugal; imalves@fe.up.pt (I.M.Q.); mob@fe.up.pt (M.O.B.); adrian@fe.up.pt (A.M.T.S.); ritalado@fe.up.pt (A.R.L.R.)

**Keywords:** tap water, micropollutants, priority substances, contaminants of emerging concern, analytical chemistry, European legislation, hazard quotients

## Abstract

Scientific concern regarding the widespread occurrence of micropollutants (MPs) in aquatic environments has been growing. **Background/Objectives**: Since conventional wastewater and drinking water (DW) treatment plants are generally unable to completely remove MPs, their presence in DW may occur, potentially posing adverse effects on public health. Highly sensitive analytical methods are crucial, as MPs may occur at very low concentrations in DW, usually at ng L^−1^ levels. **Methods**: An offline solid-phase extraction ultra-high performance liquid-chromatography coupled to tandem mass-spectrometry (SPE-UHPLC-MS/MS) method was optimized and validated for the determination of 23 MPs in DW, including 12 pharmaceuticals, 9 pesticides, and 2 UV-filters, listed in the 2 most recent European Union (EU) Decisions (2022/1307 and 2025/439) for surface water monitoring, and in the revised EU Urban Wastewater Treatment Directive (2024/3019). The validated method was applied to 50 DW samples collected across Portugal. **Results**: The optimized SPE-UHPLC-MS/MS method showed high analytical sensitivity, achieving method detection limits below 1.50 ng L^−1^. Up to 3 MPs were detected per sample, with quantifiable concentrations of each ranging from 0.28 to 98.8 ng L^−1^. However, benzotriazole and dimoxystrobin exceeded the upper limits of their calibration curves (i.e., concentrations higher than 133 and 117 ng L^−1^, respectively) in one and 3 of the collected samples, respectively. Considering all analyzed samples, 4 (fluconazole, irbesartan, dimoxystrobin, and benzotriazole) of the 23 target compounds were detected. Hazard quotient values for all detected MPs were well below 0.1. **Conclusions**: The validated SPE-UHPLC-MS/MS method is suitable for the sensitive determination of MPs in DW. Some MPs were detected, with concentrations indicating no expected human health risks under the conditions evaluated. Further monitoring campaigns should be conducted in the future, with compounds exceeding the limits of the calibration curves requiring special attention.

## 1. Introduction

Access to safe and clean drinking water (DW) is a fundamental human right [1]. However, both water scarcity and adequate water quality remain major global challenges [2]. Increasing demand driven by population growth, industrialization, and water-intensive sectors, coupled with rising wastewater production and insufficient treatment, continue to threaten freshwater availability and quality, and consequently DW production [2,3].

In recent years, growing attention has been directed towards micropollutants (MPs) in aquatic environments, which typically occur at residual concentrations in the range of ng L^−1^ to μg L^−1^ [4]. Most conventional wastewater treatment plants (WWTPs) are not yet designed to completely eliminate these compounds, leading to their discharge into freshwater bodies such as rivers and lakes, which frequently serve as sources for drinking water treatment plants (DWTPs) [4,5]. Since DWTPs also lack the capacity to completely remove most MPs at trace levels, the likelihood of their presence in tap water remains significant [6]. Indeed, the occurrence of several classes of organic MPs has been reported in DW, namely pharmaceuticals, personal care products, industrial chemicals, pesticides, fire retardants, and other substances [6,7]. Their potential effects on human health include chronic-toxicity and antimicrobial resistance; however, the extent of these impacts is not yet fully-understood, and additional effects may be identified in the future [5,8].

In response to these concerns, the European Union (EU) has progressively reinforced its regulatory framework on water quality. The Water Framework Directive (WFD; 2000/60/EC, [9]) laid the foundation for sustainable water management, followed by successive directives and decisions that introduced and updated lists of MPs, including priority substances (PSs), for which environmental quality standards are established, and contaminants of emerging concern (CECs) subject to EU-wide monitoring. Watch Lists for CECs, first implemented in 2015 [10], have undergone several revisions [11,12,13], with the latest update comprising 29 CECs (Decision 2025/439/EU, [14]). In parallel, the Drinking Water Directive (DWD; 2020/2184/EU, [15]) and the revised Urban Wastewater Treatment Directive (UWWTD; 2024/3019/EU, [16]) also address MPs, with the latter including, for the first time, 13 organic MPs for which removal rates are specified.

The occurrence of MPs in DW has been reported worldwide [17,18,19], including in Portugal [6]. However, a comprehensive study covering different regions of Portugal and a wide range of target compounds has not yet been conducted. Owing to the widespread concern regarding the presence of MPs, the development of highly sensitive, robust, and multi-residue analytical methods is essential for their determination in DW matrices.

Therefore, this study aims, for the first time, to optimize and validate a solid-phase extraction (SPE) coupled with ultra-high performance liquid-chromatography tandem mass-spectrometry (UHPLC-MS/MS) method for the analysis of a broad range of MPs in DW, and to apply it to samples collected from different network suppliers operating across Portugal. The list of target MPs includes relevant EU compounds from the fourth [13] and fifth [14] Watch Lists, as well as from the revised UWWTD [16]. The quality of Portuguese DW in terms of MPs is assessed, thereby contributing to the generation of high-quality monitoring data, in line with the EU recommendations and advancing scientific knowledge in this field. In addition, a preliminary human health risk assessment is conducted through the estimation of hazard quotients (HQs) for the detected MPs.

## 2. Results and Discussion

### 2.1. SPE Optimisation

The SPE-UHPLC-MS/MS method used as a starting point in this project was originally validated by the research group to determine MPs in surface waters [5], a matrix that does not require the use of dechlorination or chelating agents during sample preparation. However, when dealing with DW matrices, where chlorine can be present, several studies report that the use of these agents can significantly enhance MP recovery rates [6,20,21], as discussed further below. Although dedicated experiments on dechlorination-induced analyte stability were not performed, overall recoveries inherently account for potential losses, providing a practical indication that analyte integrity is maintained under the applied method conditions. Therefore, either ascorbic acid (C_6_H_8_O_6_, [6,21]) or sodium thiosulfate (Na_2_S_2_O_3_, [6,20]) was tested to assess the impact of dechlorination effects on absolute recoveries (ARs), and disodium EDTA dihydrate (Na_2_·EDTA·2H_2_O; 208 mg, [6]) was evaluated as a chelating agent.

The initial comparison consisted in examining recoveries, i.e., ARs in water samples pretreated with 2 different masses of C_6_H_8_O_6_ (9.4 mg [21] or 3.75 mg [6]), 3 different masses of Na_2_S_2_O_3_ (13.5 mg [6], 11.25 mg [6], or 30 mg [20]), and with no additives, aiming to assess the need to use any dechlorination agent and to determine the optimal mass of each agent.

The results showed that the addition of 9.4 mg of C_6_H_8_O_6_ to water samples prior to SPE yielded higher ARs for most MPs (13 out of 23) compared to both 3.75 mg and no additives. Among the 9 target pesticides, 7 exhibited improved ARs with 9.4 mg of C_6_H_8_O_6_, confirming this dose as the optimal amount of the agent. Similarly, the addition of 13.5 mg of Na_2_S_2_O_3_ yielded the highest recovery rates for 14 out of 23 compounds, outperforming the other 2 concentrations tested of the same agent (11.25 and 30 mg) as well as samples with no additives. Out of the 9 target pesticides and the 2 target UV-filters, they all achieved higher recovery rates with the use of 13.5 mg of Na_2_S_2_O_3_ in comparison to samples with different doses of the same agent or without any additive.

A final comparison assessed whether the addition of dechlorination or chelating agents (i.e., Na_2_·EDTA·2H_2_O) improved ARs relative to samples with no additives. ARs were higher for only 6 out of 23 MPs in samples without additives, indicating that the use of additives is generally preferable. Regarding the addition of Na_2_·EDTA·2H_2_O, improvements were observed for some compounds, likely due to its ability to chelate residual metals in the matrix that otherwise bind to MPs and reduce ARs [6]. However, this effect was limited, benefiting fewer than half of the target compounds (i.e., 7 MPs). Therefore, Na_2_S_2_O_3_ was selected as the optimal additive. This is likely due to the superior ability of Na_2_S_2_O_3_ to quench residual chlorine typically present in DW as a result of disinfection during the supply process [6]. Accordingly, 13.5 mg of Na_2_S_2_O_3_ was consistently added to the 375 mL of water samples prior to SPE extraction to enhance the recovery rates of the target MPs.

### 2.2. Drinking Water Method Validation

The DW method validation was performed by applying the internal calibration method as detailed in Section 3.4. Ideally, each MP would be quantified according to its respective internal standard. However, the high costs of the internal standards make this approach impractical for routine environmental monitoring. Additionally, finding suitable internal standards for each compound is challenging, especially for this type of multi-residue methods that involve a wide range of compounds with distinct properties. As a result, compounds were grouped based on their physicochemical characteristics, as shown in Table S2 (Supplementary Material), with each group assigned to a specific internal standard. This approach is consistent with other published studies on multi-class organic MPs [5,6,22], which also adopted grouping strategies based on physicochemical properties (e.g., polarity, molecular weight, or acid-base nature). Therefore, although ideally each MP would be corrected with its own isotopically labelled internal standard, grouping compounds according to physicochemical similarity, combined with matrix-matched calibration, provides a literature-supported strategy to mitigate ionization variability in multi-class ESI-MS/MS methods [5,6,22].

The parameters of the calibration curves (range, coefficient of determination (*r*^2^), instrument detection limit (IDL), instrument quantification limit(IQL), method detection limit (MDL), and method quantification limit (MQL)) for the 23 target MPs, as well as their retention times, are summarized in Table S3 (Supplementary Material). Depending on the analyte, the method achieved linearity between 5.00–40.0 ng L^−1^ and 100–133 ng L^−1^, corresponding to the lowest and highest calibration points of the obtained curves. The *r*^2^ values ranged from 0.995 to 0.999, meeting the required criteria. The instrumental limits, IDL and IQL, ranged from 0.05 to 5.69 μg L^−1^, and from 0.17 to 17.2 μg L^−1^, respectively. Regarding the method limits that consider the entire SPE-UPHLC-MS/MS analytical method, MDL and MQL ranged, respectively, from 0.02 to 1.47 ng L^−1^, and from 0.08 to 4.45 ng L^−1^.

**Figure 1:**
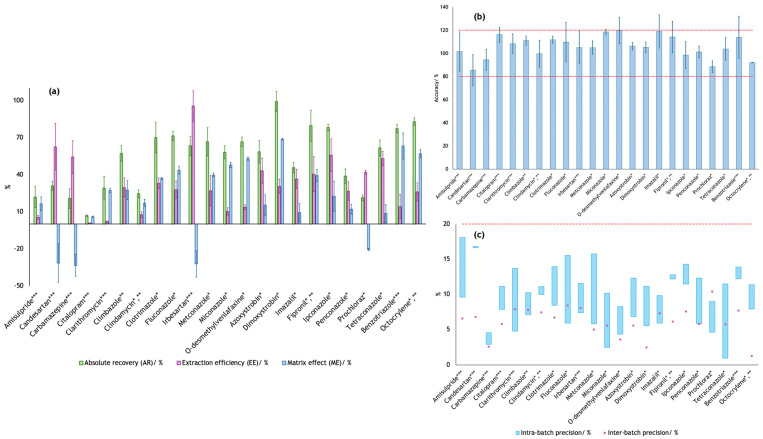
ARs, EEs, MEs (**a**), accuracies (**b**), and intra- and inter-batch precisions (**c**) for each of the 23 target MPs from Watch List 2022 (*) [13] and 2025 (**) [14], and from the revised UWWTD (***) (Directive 2024/3019/EU) [16]. Threshold values set by international guidelines [23] are illustrated by the red dashed lines in (**b**,**c**).

In addition to the parameters listed in Table S3 (Supplementary Material), ARs, extraction efficiencies (EEs), matrix effects (MEs), accuracies, and intra- and inter-batch precisions were also evaluated for each MP. These results are shown in Figure 1 (exact values are summarized in Table S4 (Supplementary Material)).

The average AR obtained was 53.4%, with dimoxystrobin showing the highest AR (99.1%), and citalopram the lowest (6.74%). Although citalopram exhibited a considerably low AR (the only MP with an AR lower than 10%), the method showed high repeatability for this compound, with a low relative standard deviation (5.8%, RSD), indicating good precision. Low but consistent recoveries are common in multi-residue UHPLC-MS/MS methods. When recoveries are reproducible, MEs are adequately corrected, and responses remain linear within the validated range, quantitative results can still be considered reliable [6,22], although concentrations should be interpreted with caution. Since the AR considers the efficiency of the entire analytical process, it should be interpreted together with the EE and ME. The average EE was 32.0%, with candesartan being the MP with the highest EE (62.4%), and citalopram the one with the lowest EE. A general examination of Figure 1a clearly shows that the obtained values of AR, EE, and ME differ considerably between the compounds, which is expected in multi-residue methods [6,22]. Interestingly, the average AR of the UV-filters (~80%) is higher than the average AR of both pesticides (~61%) and pharmaceuticals (~43%). However, the average EE of the UV-filters (~20%) is the lowest of the 3 classes of MPs—pharmaceuticals have an average EE of ca. 30%, and pesticides of ca. 38%. This suggests that UV-filters are more susceptible to enhancement matrix effects than the other classes of MPs, a topic that is explored further below. ME must be carefully evaluated, as it can easily lead to misinterpretations. For instance, a compound exhibiting exceptionally high AR does not necessarily indicate high EE during SPE; in some cases, substantial signal enhancement caused by the matrix may mask poor EE, or conversely, signal suppression may obscure an otherwise high AR. This behavior was observed for most of the target compounds: only 3 MPs—citalopram, imazalil, and tetraconazole—showed similar AR and EE values (i.e., within ± 10%), suggesting that for 20 out of 23 compounds the signal was significantly affected by the impact of the matrix constituents during the analytes’ ionization. For example, as previously mentioned, dimoxystrobin had the highest AR (99.1%); however, it also exhibited the most pronounced positive ME (68.6%), which explains its low EE (30.5%), and demonstrates that high ARs do not necessarily correspond to high EEs. Regarding positive MEs, the average was 32.1%, which is substantially high, with values ranging from 5.9% (citalopram) to 68.6% (dimoxystrobin). Similarly, the average of the negative MEs was −29.5%, ranging from −33.5% (carbamazepine) to −20.7% (prochloraz). Interestingly, both target UV-filters showed MEs higher than 55%, which proves what was previously anticipated—UV-filters are highly influenced by the effect that the naturally present components in the sample matrix have on the ionization source [24]. Despite the occurrence of pronounced MEs for most MPs, full validation in the target matrix combined with the use of internal standards ensures reliable quantification. This strategy is widely accepted in multi-residue UHPLC-MS/MS methods when compound-specific isotopically labeled standards are not feasible for each analyte, and grouping strategies must be implemented [5,6].

Considering this analysis, Figure 1a allows for clearer evaluation of whether the observed AR is mainly influenced by signal enhancement or suppression (i.e., ME), or if it aligns with the EE, in this case the matrix having a negligible effect on the compounds’ signal detection. It is important to note that lower ARs and pronounced MEs are commonly observed in multi-residue methods involving chemically diverse analytes in complex matrices and do not necessarily compromise quantitative reliability when adequate precision, linearity, and sensitivity are demonstrated [22]. The data clearly indicate that DW is a complex matrix that may lead to substantial signal enhancement or suppression for many MPs.

Regarding the accuracy results obtained for each target MP, according to European guidelines [23], recommended accuracy values should fall within the range of 80 to 120%, suggesting agreement between the obtained quality control (QC) results and the theoretical values. This criterion was met by all 23 target MPs, as shown in Figure 1b. In addition, the method’s precision was evaluated through both intra- and inter-batch precision. According to recent international standards [25], the RSD should remain below 20%, which was successfully achieved for all 23 target MPs (Figure 1c).

Finally, results regarding the method’s evaluation using the multi-color assessment platform for white analytical chemistry (MA tool, [26]) are summarized in Figure S1 (Supplementary Material), demonstrating a final whiteness score of 55.1%, reflecting moderate overall performance.

### 2.3. Portuguese Drinking Water Sample Analysis

The optimized and validated SPE-UHPLC-MS/MS method was applied to monitor the occurrence of 23 target MPs in 50 DW samples collected from domestic taps across Portugal, providing a representative overview of diverse geographical areas and potential influencing factors. Table 1 summarizes the concentration ranges observed for each of the 23 target MPs across all samples, together with their corresponding detection frequencies. Tables S5 and S6 (Supplementary Material) report the concentrations of each MP in each individual sample, along with the propagated uncertainty from analytical variability, expressed for each MP detected at a quantifiable concentration in each sample.

Out of the 23 target MPs, only 4 were detected. It is important to mention that in Portugal, especially in rural areas, wells are sometimes used as the main water source, and although such water should be used only for irrigation and non-potable domestic uses, sometimes it is used for human consumption without checking the quality requisites.

**Figure 2:**
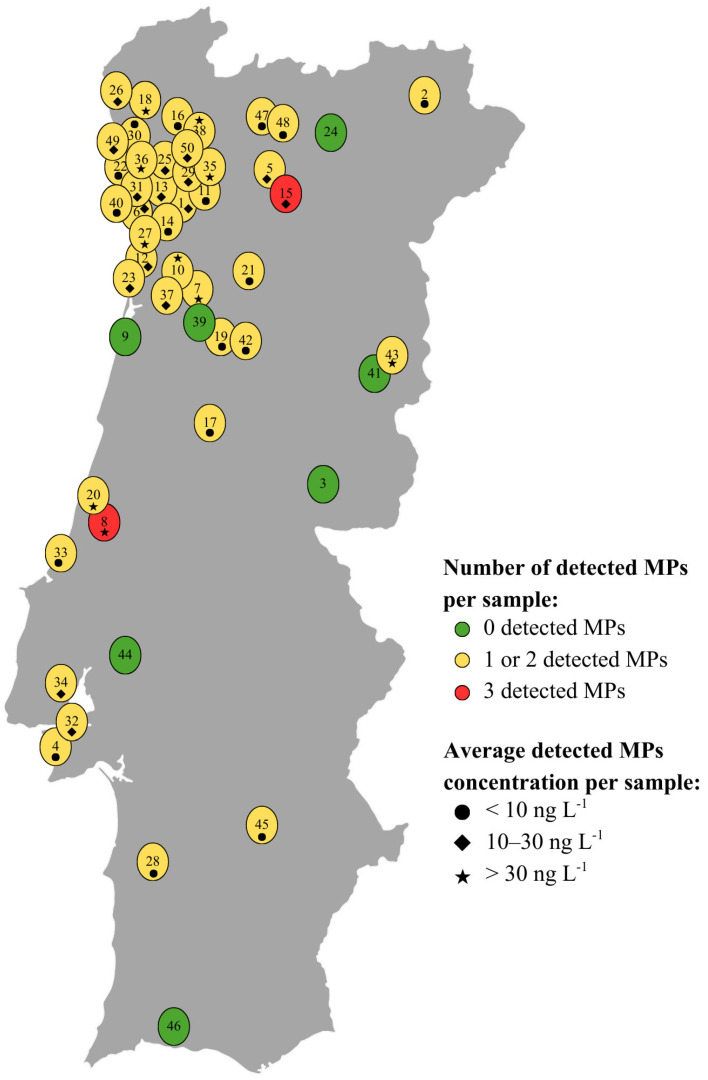
Approximate detection frequency of each MP (represented in green, yellow, or red) and average detected concentration range (represented by a circle (●), lozenge (◆), or a star (★)) in each of the 50 analyzed DW samples (numbered from 1 to 50 according to collection order).

Among the 12 target pharmaceuticals, only irbesartan and fluconazole were detected, with frequencies of 3 and 2 samples, respectively, out of a total of 50 samples. This was expected considering that the source of these compounds is related to their presence in surface and groundwaters that receive diffuse pollution, mainly from effluents of WWTPs, where such compounds may not be fully degraded [7]. The maximum detected concentration of irbesartan was very low (2.43 ng L^−1^ in sample 15), as were the concentrations measured in the other 2 samples in which it was detected (0.37 ng L^−1^ in sample 8 and 0.28 ng L^−1^ in sample 14). In contrast, fluconazole was detected at a low concentration in sample 12 (3.88 ng L^−1^), but at a markedly higher concentration in sample 15 (38.4 ng L^−1^). Notably, sample 15 was the only sample in which both pharmaceuticals were detected, and in both cases at their respective determined maximum concentrations. Although based on a limited number of detections, sample 15—in which the highest concentrations were detected—was collected from an inland region of Portugal, whereas the remaining positive samples were located in coastal areas, as further summarized in Figure 2.

Regarding the 9 target pesticides, only dimoxystrobin was detected among the collected samples. This compound was detected in 9 out of 50 samples at quantifiable concentrations ranging from 6.31 ng L^−1^ (sample 2) to 37.1 ng L^−1^ (sample 8). Additionally, in 2 samples (samples 11 and 19), dimoxystrobin was detected at concentrations below its MQL (0.61 ng L^−1^), while in 3 samples (samples 18, 43, and 50) it was detected at concentrations that fall outside the range covered by the respective calibration curve, i.e., at concentrations that exceeded 117 ng L^−1^. Dimoxystrobin occurrence in tap water may result from agricultural runoff, thus reaching DW sources.

A similar situation was observed for benzotriazole, which was the only UV-filter detected among the 2 target compounds of this class. This compound is a very persistent corrosion inhibitor [5] and may be originated from plumbing and distribution systems. Benzotriazole exhibited the highest and most concerning detection frequency, being detected in 43 out of 50 samples, with quantifiable concentrations between 0.95 ng L^−1^ (sample 45) and 98.8 ng L^−1^ (sample 20). Furthermore, in one sample (sample 47), it was detected at a concentration below its MQL (0.19 ng L^−1^), and in one sample (sample 38), at a concentration that exceeds the upper limit of its calibration curve, i.e., at a concentration higher than 133 ng L^−1^. Given the nature of the studied matrix (i.e., DW), such high concentrations were not anticipated when validating the method and, therefore, the calibration curves did not include points to account for these cases.

It is important to note that, for the 2 MPs that were detected in some samples at concentrations exceeding the upper limits of their calibration curves, results were conservatively treated as semi-quantitative: the actual concentration may be higher than the highest calibration point but cannot be accurately quantified due to the method not being validated in that range. Ideally, those calibration curves would be extended to include higher concentration levels to enable full quantification of the samples; this aspect will be addressed in future method optimization.

Notably, the available literature indicates that certain MPs, such as dimoxystrobin, have not been previously investigated in DW and have been studied only in surface water matrices. Thus, to the best of our knowledge, this is the first work that simultaneously investigates and reports the presence of this MP in DW matrices. In this study, dimoxystrobin was detected in 9 of the 50 samples, with concentrations in 3 samples exceeding the upper limit of the calibration curve.

Figure 2 provides an illustrative overview of the number of MPs detected per sample and their respective average concentration ranges. For MPs detected at concentrations above the respective calibration curve’s upper limit, the highest calibration point was used. For compounds detected at concentrations below its MQL, the respective MQL value was used for calculating the average detected concentration in each sample in order to account for a worst-case scenario.

Regarding the number of detected MPs per sample, out of 50 collected samples, only 7 showed no detectable MPs among the 23 target compounds. Notably, these 7 samples were the only ones in which benzotriazole was not detected, as this MP exhibited the highest detection frequency (43 out of 50 samples). A total of 62% of samples (31 out of 50) contained only one detected MP, which was the UV-filter benzotriazole in all cases. Furthermore, 10 and 2 samples contained 2 and 3 MPs, respectively, and no samples contained all 4 detected MPs together.

Regarding the average concentrations of the detected MPs per sample, 10 samples had concentrations above 30 ng L^−1^. From these, 4 samples corresponded to the cases in which either benzotriazole or dimoxystrobin were detected at concentrations exceeding the upper limit of their calibration curves. The remaining 6 samples all had average concentrations of the detected MPs above 30 ng L^−1^ due solely to the presence of benzotriazole. Furthermore, 17 samples showed average concentrations below 10 ng L^−1^, while the remaining 16 samples had average concentrations between 10 and 30 ng L^−1^.

Considering both factors simultaneously, i.e., the number of MPs detected per sample and their respective average concentration ranges, almost all samples containing either one or 2 MPs have an average detected MPs concentration below 30 ng L^−1^ (32 out of 41 samples), with only 9 samples exceeding this value. Regarding the 2 samples with 3 detected MPs, one had an average concentration within the 10 to 30 ng L^−1^ range, while sample 8 was the most concerning, containing three MPs and an average detected MPs concentration of 31.1 ng L^−1^.

The data showed no clear trend. This suggests that any DW sample collected from the Portuguese taps was likely to contain a similar number of MPs at comparable average concentrations, regardless of its origin. In addition, the number of analyzed samples and the low detection frequency for many MPs indicate that these results cannot be generalized to represent the entire national DW system. While some consistency was observed in the concentrations of the detected compounds, further studies with larger sample sizes, broader geographic coverage, and sampling across different seasons to account for seasonal variations are needed to confirm these preliminary findings and support any national-level conclusions regarding DW safety, as well as the performance of Portuguese WWTPs and DWTPs in removing these target MPs. Additionally, the detection of 2 MPs at concentrations exceeding the upper limit of the calibration curve in at least one sample further highlighted the need for continued and expanded monitoring efforts, as well as studies on analysis without preconcentration using UHPLC-MS/MS.

### 2.4. Hazard Quotients: Human Health Risk Assessment

A preliminary human health risk assessment was performed to evaluate the potential risk associated with the concentrations of MPs detected in the 50 DW samples. This was accomplished through the estimation of a HQ for each individual substance in each DW sample. It is important to mention that HQs were evaluated considering the analytical uncertainty associated with each MP concentration, and calculations performed using both the lower and upper uncertainty bounds did not modify the overall HQ range obtained.

The average daily intake (ADI) values for the detected compounds were retrieved from the literature, namely for irbesartan and fluconazole [27], dimoxystrobin [28], and benzotriazole [29]. It is important to note that for MPs detected below the MQL, this value was applied to represent a worst-case scenario. Conversely, for compounds detected at concentrations above the upper limit of their respective calibration curve, the highest calibration point was used, which was the only feasible approach, although it may underestimate the actual risk. However, to evaluate the magnitude of potential underestimation, a sensitivity analysis was conducted by doubling the highest calibration point values. The resulting HQs remained below thresholds of concern, confirming the robustness of the risk assessment.

HQ values help to assess the probability of adverse health effects: HQ values below 0.1 indicate no expected adverse effects; values between 0.1 and 1.0 suggest potential for adverse effects that, while unlikely, should not be disregarded; HQ values ranging from 1.0 to 10 indicate adverse effects or a mild risk; and values above 10 are considered to represent a high risk [6]. The concentrations detected in the 50 DW samples targeting individual MPs do not suggest any relevant health risks (i.e., HQ < 0.1) for either adults or children. However, it is important to note that these HQs were evaluated for each individual substance per sample, which does not reflect real conditions—12 out of 50 samples exhibited more than a single MP. Therefore, although these HQs are quite low, they do not account for potential mixture (cocktail) effects arising from the simultaneous presence of multiple MPs in each sample. This model has limitations since it does not consider uncertainties in exposure, toxicity, and the potential for synergistic effects [6], which can only be overcome by more robust risk assessment models that are not yet available, for instance, predicting the long-term health impacts of exposure to multiple MPs. Although these HQs were calculated on a single-compound basis and therefore do not account for potential mixture effects, this approach provides a conservative and appropriate framework for preliminary DW risk screening, with more refined cumulative assessments to be explored in future work. In addition, it should be noted that the HQs were calculated based on an average adult or child scenario for most compounds. Consequently, the assessment does not explicitly consider other vulnerable populations such as pregnant women or other high-exposure groups, which may experience higher relative exposures.

## 3. Materials and Methods

### 3.1. Chemicals and Materials

In this work, 23 MPs from various classes listed in the 2 most recent EU Watch Lists (2022 and 2025) and in the UWWTD were studied, comprising: 12 pharmaceuticals and metabolites (amisulpride, candesartan, carbamazepine, citalopram, clarithromycin, climbazole, clindamycin, clotrimazole, fluconazole, irbesartan, miconazole, and o-desmethylvenlafaxine); 9 pesticides (azoxystrobin, dimoxystrobin, fipronil, imazalil, ipconazole, metconazole, penconazole, prochloraz, and tetraconazole); and 2 organic UV-filters (benzotriazole, and octocrylene). Table S1 (Supplementary Material) summarizes the class, subclass, chemical structure, molecular weight (M*_w_*), and p*K*a of all analytes.

All 23 reference standards (>98% purity) and all 6 internal standards (carbamazepine-*d*_10_, ciprofloxacin-*d*_8_, citalopram-*d*_6_, ofloxacin-*d*_3_, prochloraz-*d*_4_, venlafaxine-*d*_6_; >98% purity) were purchased from Sigma–Aldrich (Steinheim, Germany). Individual stock solutions of each reference and internal standard were prepared in acetonitrile (ACN) by dissolving known amounts of each standard to achieve concentrations ranging approximately from 500 to 1000 mg L^−1^. In addition, a solution containing all reference standards and another containing all internal standards were prepared at concentrations of 2.5 mg L^−1^ and 1.0 mg L^−1^, respectively, by dilution of the individual solutions in ACN. These solutions were used to spike DW and ultrapure water (UPW) samples during the optimization and validation of the SPE-UHPLC-MS/MS method.

Ethanol (EtOH; HPLC grade) was purchased from Fisher Scientific (Leicestershire, UK), while methanol (MeOH) and ACN, both MS grade, were obtained from VWR International (Fontenay-sous-Bois, France). Formic acid was purchased from Merck Sharp & Dohme (Darmstadt, Germany) and sulfuric acid was supplied from VWR International (Fontenay-sous-Bois, France). Na_2_S_2_O_3_ (99% purity) and C_6_H_8_O_6_ (99% purity) were purchased from Sigma-Aldrich (Steinheim, Germany), and Na_2_·EDTA·2H_2_O (99% purity) was acquired from ITW Reagents Panreac (Spain). UPW was supplied by a Milli-Q SQ 2Series water system (resistivity > 18.2 MΩ cm at 25 °C) (Burlington, MA, USA). A multiparameter inoLab^®^ probe (WTW, Frankfurt am Main, Germany) was used for pH adjustments. Oasis^®^ HLB (Hydrophilic-lipophilic balanced) SPE cartridges (150 mg, 6 mL) were purchased from Waters^TM^ (Milford, MA, USA).

### 3.2. Solid-Phase Extraction Protocol

Tap samples were used to optimize and validate the SPE-UHPLC-MS/MS method previously developed by our research group for monitoring MPs in surface water matrices [5]. SPE experiments were always carried out in triplicate using a 20-position extraction manifold supplied by Waters^TM^ (Milford, MA, USA). Oasis HLB cartridges (150 mg, 6 mL) were used with no conditioning required, as demonstrated by prior tests conducted by the research group [5], and as supported by the manufacturer, which states that these cartridges allow direct sample loading, providing reliable recoveries while simplifying the workflow [30]. Briefly, as described elsewhere [5], 375 mL of each sample was adjusted to pH 3 using sulfuric acid (MS grade; 94% purity) for acidification. After sample loading, the cartridges were washed with 4 mL of UPW to ensure the desorption of most polar interferents, then dried for 45 min under vacuum conditions using a pump (VWR International) connected to the extraction manifold. The elution was performed with 3.5 mL of EtOH and although the elution solvent was not specifically optimized for nonpolar analytes, method development described elsewhere [5] demonstrated acceptable results across all target compounds, reflecting standard practice in broad-spectrum environmental SPE-UHPLC-MS/MS methods. The extract was subsequently dried in a CentriVap^®^ Concentrator (LABCONCO, Kansas City, MO, USA) until complete evaporation. Finally, the dried extracts were reconstituted in 250 μL of a 80/20 (*v*/*v*) MeOH/UPW solution, and the resulting reconstituted extracts were filtered using 0.22 μm polytetrafluoroethylene (PTFE) hydrophobic syringe filters (Membrane Solutions, Auburn, TX, USA). Due to the possible presence of chlorine in DW samples, the addition of a dechlorination agent—Na_2_S_2_O_3_ (13.5 mg [6], 11.25 mg [6], and 30 mg [20]) or C_6_H_8_O_6_ (9.4 mg [21] and 3.75 mg [6])—or a chelating agent—Na_2_·EDTA·2H_2_O (208 mg [6]), to the initial sample was additionally tested.

### 3.3. Liquid-Chromatography Tandem Mass-Spectrometry Analysis

The UHPLC-MS/MS analysis was performed using an integrated apparatus from Shimadzu Corporation (Tokyo, Japan), which combines LC and tandem MS detection. The LC system comprises a Nexera ultra-high-performance analytical chromatography (UHPLC) module equiped with 2 LC-30AD pumps, a SIL-30AC autosampler, a CTO-20AC oven, a DGU-20A 5R degasser, and a CBM-20A system controler equiped with LC Solution Version 5.41SP1 Software. The MS detector includes a triple quadrupole (QqQ) mass spectrometer detector LCMS-8040 Ultra-Fast Mass-Spectrometry, with electrospray ionisation.

The LC method applied was based on a previous work of the research group [5]. A Kinetex™ 1.7 μm F5 (pentafluorophenyl) 100 Å (100 × 2.1 mm, i.d.) chromatographic column (Phenomenex, Inc.,Torrance, CA, USA) was employed. The mobile phases consisted of UPW and MeOH, both acidified with 0.1% of formic acid, at a flow rate of 0.20 mL min^−1^ under gradient mode. The gradient ramp began with 5% MeOH for 0.2 min, followed by a linear increase from 5% to 98% MeOH over 29.8 min, which was maintained for 2.0 min. Posteriorly, a rapid decrease to 5% MeOH occurred within 0.1 min, followed by re-equilibration for 3.9 min, resulting in a total run of 36.0 min. The column oven and autosampler temperatures were maintained at 30 °C and 15 °C, respectively, and the injection volume was 10 μL.

MS analysis was performed with multiple reaction monitoring (MRM) for quantification, using the MS parameters (electrospray ionization mode, decluttering potential, collision energy, and collision cell exit potential) described in Table S2 (Supplementary Material). Electrospray ionization under negative ionization mode was applied to 3 MPs, with the molecular ion [M-H]^-^, whereas the other 20 MPs and all internal standards were ionized under positive ionization mode, with the molecular ion being either [M+H]^+^ or [M+Na]^+^. The most intense transition (MRM1) was selected for quantification, while the retention time and the ratio between transitions (MRM1/MRM2) were used to confirm compound identity. The instrument was operated with a capillary voltage of 0.5 kV, a drying gas flow of 14 L min^−1^, a nebulizing gas flow of 2.8 L min^−1^, and desolvation and source temperatures of 250 and 400 °C, respectively, replicating the conditions described in the original method [5]. The collision induced dissociation gas was argon at 230 kPa.

### 3.4. Method Validation

The SPE-UHPLC-MS/MS method was validated according to international guidelines [23] and other relevant studies on the topic [5,22], considering the following parameters: selectivity, linearity, range, precision, accuracy, AR, EE, ME, MDL and MQL. Additionally, an evaluation of the proposed method was performed using the multi-color assessment platform for white analytical chemistry (MA tool, [26]) to ensure consistency, validity, and objective assessment of the analytical performance, greenness, practicality, and innovation.

Selectivity was evaluated by comparing the chromatograms of the target analytes in a MeOH/UPW solution (80/20, *v*/*v*) with those of the same compounds in the extracts of spiked DW samples. The internal standard calibration method, using triplicate matrix-matched curves, was applied to determine the linearity range of each MP. For that, DW samples were spiked at 12 different analyte concentrations: 5.00, 10.0, 20.0, 30.0, 40.0, 50.0, 60.0, 73.3, 83.3, 100, 117, and 133 ng L^−1^. Additionally, 40 μL of an internal standard solution at 1 mg L^−1^ was added to each sample. A volume of 375 mL of the DW samples was processed by SPE and reconstituted in 250 μL of a MeOH/UPW solution (80/20, *v*/*v*), corresponding to a concentration factor (*f*) of 1500. It is important to mention that while formal residual and lack-of-fit analysis were not performed, linearity was confirmed through r^2^ values higher than 0.995, matrix-matched QC recoveries, and repeated control injections, providing reliable quantitative performance consistent with standard LC-MS/MS validation practices for multi-residue environmental analysis [5,22]. 3 QCs, with DW spiked concentrations of 17.0, 57.0 and 80.0 ng L^−1^, were processed on 3 different days for determination of intra- and inter-batch precisions. Precision was expressed as the RSD derived from the triplicate measurements of the QCs, either within the same day or between 3 different days, depending on whether it regards intra- or inter-batch precision, respectively. For accuracy determination, the percentage of agreement between the concentrations determined in the SPE extracts and their nominal concentrations was evaluated.

**Figure 3:**
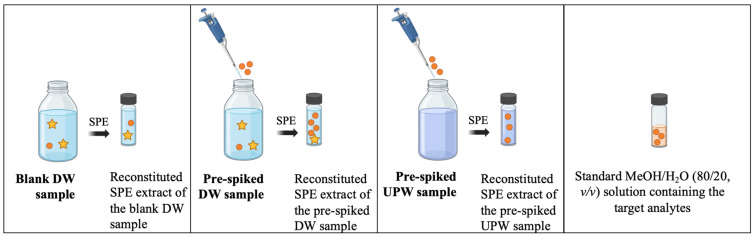
Schematic representation of the process for calculating the AR, EE, and ME percentages (Adapted from [5]). Circles (●) represent target analytes and stars (★) represent possible interferents.

To aid interpretation, Figure 3 illustrates the determination of the AR, EE, and ME percentages, along with the corresponding equations (Equations (1)–(3), respectively) described in the text.

The AR considers the entire analytical process. It measures the global efficiency of the SPE-UHPLC-MS/MS method for each target substance, taking into consideration both contributions of the SPE recovery (i.e., the EE) and the ME. Therefore, peak areas of the analytes present in the reconstituted SPE extracts of pre-spiked samples—to which the peak areas of the analytes present in the blank samples were subtracted—were compared to the peak areas of the analytes in a MeOH/H_2_O (80/20, *v*/*v*) solution containing the target analytes at a concentration theoretically equal to the one of the reconstituted SPE extracts (Equation (1)).

(1) $$AR\;\left(\%\right)=\frac{Area_{\mathrm{pre}-\mathrm{spiked}\;\mathrm{DW}\;\mathrm{extract}}-Area_{\mathrm{blank}\;\mathrm{DW}\;\mathrm{extract}}}{Area_{\mathrm{standard}\;\mathrm{solution}}}\times 100$$

The EE of the analytes during the SPE procedure was calculated as the ratio between the peak areas of analytes in the reconstituted SPE extracts of pre-spiked UPW samples with the peak areas of the analytes in a MeOH/H_2_O (80/20, *v*/*v*) solution containing the target analytes at a concentration theoretically equal to the one of the reconstituted SPE extracts (Equation (2)).

(2) $$EE\;\left(\%\right)=\frac{Area_{\mathrm{pre}-\mathrm{spiked}\;\mathrm{UPW}\;\mathrm{extract}}}{Area_{\mathrm{standard}\;\mathrm{solution}}}\times 100$$

The ME of the analytes was calculated by subtracting the EE from the AR (Equation (3)), which is equivalent to comparing the peak areas of the analytes in the reconstituted SPE extracts of pre-spiked DW samples with the peak areas of the analytes in the reconstituted SPE extracts of pre-spiked UPW samples. Therefore, it explains the effect that the naturally present components in the sample matrix have on the ionization source. Negative ME values indicated that the DW caused ion suppression and a loss of analyte MS signal, whereas positive ME values indicated ion enhancement in the MS signal. Despite other methodologies for determining MEs are common, such as the post-extraction spiking approach, both are widely recognized and accepted for evaluating MEs in LC-MS/MS analysis and were cross-verified with matrix-matched calibration [5,21].

(3) $$ME\;\left(\%\right)=AR-EE$$

Note that a solution in ACN containing all reference and internal standards at 500 μg L^−1^ was continuously analyzed and used as a control solution throughout the study to monitor potential discrepancies in peak intensities. Furthermore, during the UHPLC-MS/MS analysis, an injection of MeOH/UPW (80/20, *v*/*v*) was carried out between each sample injection to account for any carry-over effect, i.e., the possible accumulation of MPs in the column.

MDL and MQL values were directly obtained through the LabSolutions™ LCMS Software, while IDL and IQL were calculated by multiplying the MDL or MQL by the concentration factor and then dividing by the AR (Equations (4) and (5), respectively). Although experimental verification of MDL and MQL values through replicate low-level spiking is also common practice, in this study these limits were software-derived from matrix-matched calibration data; however, their determination under these conditions provides realistic method performance estimates for trace-level monitoring, in line with previously published multi-residue approaches [5,6].

(4) $$IDL=\frac{MDL\times f}{\frac{AR\;\left(\%\right)}{100}}$$

(5) $$IQL=\frac{MQL\times f}{\frac{AR\;\left(\%\right)}{100}}$$

### 3.5. Sample Analysis

**Figure 4:**
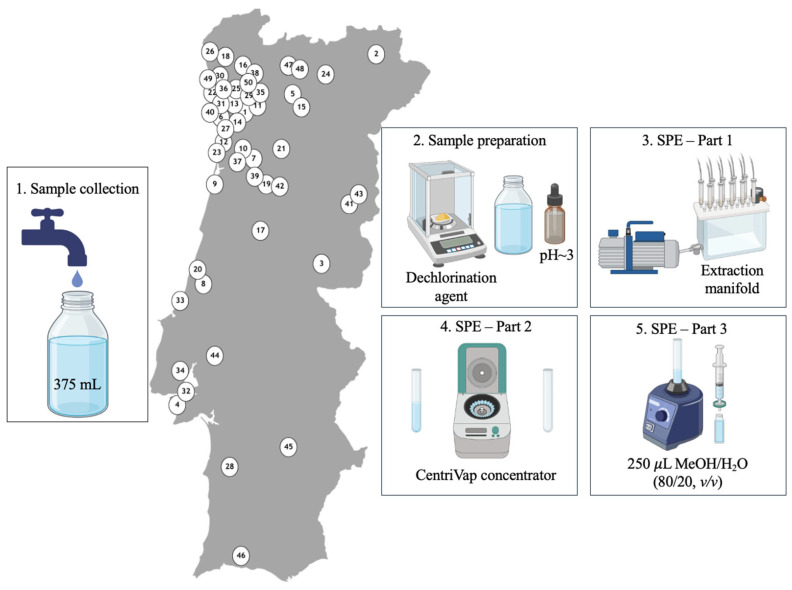
Approximate sampling locations in Portugal (numbered from 1 to 50 according to collection order) and schematic representation of the sample collection, preparation, and extraction steps.

Following thorough validation, the SPE-UHPLC-MS/MS analytical method was applied to tap DW matrices collected from numerous sites across Portugal during May and June 2025. A total of 50 samples were obtained—30 from the northern, 13 from the central, and seven from the southern regions of the country. Samples were collected directly from household taps of residents who reported being supplied by the public DW distribution system. Nevertheless, the use of private wells or boreholes as alternative or supplementary sources cannot be ruled out. Samples were collected directly into pre-rinsed glass water bottles in which they were stored under refrigeration during transportation and in the laboratory until being processed. Figure 4 illustrates the approximate sampling locations across the country—numbered from 1 to 50 according to collection order—as well as the schematic representation of the experimental steps performed until UHPLC-MS/MS analysis (Figure 2).

### 3.6. Human Health Risk Assessment

For those substances determined in DW, a preliminary human health risk assessment was performed. This was accomplished through the estimation of a HQ for each individual substance in each DW sample, following previous studies [6]. The HQ is calculated by dividing the estimated daily intake (EDI) by the ADI (Equation (6)).

(6) $$HQ=\frac{EDI}{ADI}$$

The EDI values were calculated based on the detected concentration of each MP in each of the 50 samples, considering an average body weight of 70 kg for adults and a daily water intake of 2 L day^−1^, as well as an average body weight of 15 kg for children and a daily water intake of 1 L day^−1^ [6] (Equation (7)).

(7) $$EDI=\frac{\mathrm{Detected}\;\mathrm{concentration}\;\times \mathrm{Daily}\;\mathrm{water}\;\mathrm{intake}}{\mathrm{Average}\;\mathrm{body}\;\mathrm{weight}}$$

The ADI values where retrieved from the literature or relevant databases, as described in Section 2.4.

## 4. Conclusions

A multi-residue analytical methodology of SPE-UHPLC-MS/MS was optimized and fully validated to assess the occurrence of 23 EU-relevant organic MPs in DW.

Method optimization from a previously developed protocol for surface waters was required, particularly regarding chelating and dechlorination agents. The addition of Na_2_S_2_O_3_ (13.5 mg) to the DW samples significantly improved the recoveries of more than half the target MPs (12 out of 23), compared with other agents (C_6_H_8_O_6_ and Na_2_·EDTA·2H_2_O) or no additives. The method was then validated following international guidelines, evaluating parameters such as selectivity, linearity, AR, EE, ME, and detection and quantification limits. Results for AR (mean 53.4%), EE (mean 32.0%), and ME (positive mean 32.1%; negative mean −29.5%) confirmed that DW is a complex matrix, causing significant signal enhancement or suppression, depending on the compounds. Only citalopram, imazalil, and tetraconazole showed ME values within ±10%.

Application of the method to 50 DW samples collected from different regions in Portugal revealed the presence of 4 out of the 23 target MPs (up to 3 MPs per positive sample). It is important to note that potential contamination may arise either at the source or within the distribution network; therefore, the results cannot be assumed to represent the entire national DW system. Although concentrations did not exceed 131 ng L^−1^, 2 compounds (dimoxystrobin and benzotriazole) were found above the upper limit of their calibration curves, which was unexpected and concerning in DW matrices. HQs calculated for detected MPs in DW samples indicated no expected individual risk to human health.

Overall, this study provides high-quality data for the multi-class monitoring of MPs in DW. The findings fill major research gaps in Portugal, where data remain scarce, and support EU recommendations for enhanced surveillance. They also underscore the urgent need for expanded monitoring programs and preventive measures to improve management of MPs in water intended for human consumption.

## Figures and Tables

**Table 1:** Concentration range and detection frequency for each of the 23 target MPs from Watch List 2022 (*) [13] and 2025 (**) [14], and from the revised Urban Wastewater Treatment Directive (***) (Directive 2024/3019/EU [16]). The underlined analytes were detected, in at least one sample, at concentrations higher than the upper limit of the corresponding calibration curve—Data retrieved from Tables S5 and S6 (Supplementary Material). n.d.: “not detected”.

Class	Subclass	Analyte	Range (ng L^−1^)	Frequency	Observations
Pharmaceutical	Antibiotic	Clarithromycin ***	n.d.	0/50	-
		Clindamycin *^,^**	n.d.	0/50	-
	Antidepressant	Citalopram ***	n.d.	0/50	-
		O-desmethylvenlafaxine *	n.d.	0/50	-
	Antipsychotic	Amisulpride ***	n.d.	0/50	-
	Anticonvulsant	Carbamazepine ***	n.d.	0/50	-
	Antihypertensive	Candesartan ***	n.d.	0/50	-
		Irbesartan ***	n.d.–2.43	3/50	-
	Fungicides	Climbazole **	n.d.	0/50	-
		Clotrimazole *	n.d.	0/50	-
		Fluconazole *	n.d.–38.4	2/50	-
		Miconazole *	n.d.	0/50	-
Pesticide	Insecticides	Fipronil *^,^**	n.d.	0/50	-
	Fungicide	Azoxystrobin *	n.d.	0/50	-
		Dimoxystrobin *	n.d.–37.1	9/50	3/50 samples have concentrations above the upper limit of the calibration curve for this MP, i.e., higher than 117 ng L^−1^
		Imazalil *	n.d.	0/50	-
		Ipconazole *	n.d.	0/50	-
		Metconazole *	n.d.	0/50	-
		Penconazole *	n.d.	0/50	-
		Prochloraz *	n.d.	0/50	-
		Tetraconazole *	n.d.	0/50	-
UV-filter	-	Benzotriazole ***	n.d.–98.8	43/50	1/50 sample has a concentration above the upper limit of the calibration curve for this MP, i.e., higher than 133 ng L^−1^
		Octocrylene *^,^**	n.d.	0/50	-

## Data Availability

The original contributions presented in this study are included in the article/Supplementary Material. Further inquiries can be directed to the corresponding author(s).

## Supplementary Materials

The following supporting information can be downloaded at: https://www.mdpi.com/article/10.3390/ph19030402/s1, Table S1. Target compounds selected from the EU Watch List 2022 (*) and 2025 (**), and from the revised Urban Wastewater Treatment Directive (***) (Directive 2024/3019/EU); Table S2. Multiple reaction monitoring (MRM) instrument parameters for tandem mass spectrometry analysis of each target analyte selected from Watch List 2022 (*) and 2025 (**), and from the revised Urban Wastewater Treatment Directive (***) (Directive 2024/3019/EU); Table S3. Retention time, range, linearity, instrument and method detection, and quantification limits (IDL, IQL, MDL, and MQL) for the 23 target MPs from Watch List 2022 (*) and 2025 (**), and from the revised Urban Wastewater Treatment Directive (***) (Directive 2024/3019/EU); Table S4. Absolute recovery, extraction efficiency, matrix effect, accuracy, and intra- and inter batch precision for the 23 target MPs from Watch List 2022 (*)and 2025 (**), and from the revised Urban Wastewater Treatment Directive (***) (Directive 2024/3019/EU); Table S5. Concentration in each sample (numbered from 1 to 50) of all 12 target pharmaceuticals. Quantifiable concentrations, i.e., those above the MQL, are highlighted in green and those below the MQL in yellow along with the corresponding MQL value. (n.d.: “not detected”); Table S6. Concentration in each sample (numbered from 1 to 50) of all nine target pesticides and two target UV filters. Quantifiable concentrations, i.e., those above the MQL, are highlighted in green, those below the MQL in yellow along with the corresponding MQL value, and those above the upper limit of its calibration curve in red along with its value. (n.d.: “not detected”); Figure S1. MA whiteness assessment score of the SPE-UHPLC-MS/MS method developed to determine 23 MPs in DW.

## List of Acronyms and Abbreviations

ACN: acetonitrile; ADD: average daily dose; ADI: average daily intake; AF: assessment factor; AR: absolute recovery; CEC: contaminant of emerging concern; DW: drinking water; DWD: drinking water directive; DWTP: drinking water treatment plant; EC: European Commission; EDC: endocrine disrupting compound; EDI: estimated daily intake; EDTA: ethylenediaminetetraacetic acid; EE: extraction efficiency; ESI: electrospray ionisation; EtOH: ethanol; EU: European Union; *f*: concentration factor; HLB: hydrophilic-lipophilic balanced; HQ: hazard quotient; IDL: instrument detection limit; IQL: instrument quantification limit; LC: liquid-chromatography; MDL: method detection limit; ME: matrix effect; MeOH: methanol; MQL: method quantification limit; MS: mass-spectrometry; MS/MS: tandem mass-spectrometry; MP: micropollutant; MRM: multiple reaction monitoring; *m*/*z*: mass-to-charge ratio; PS: priority substance; PTFE: polytetrafluoroethylene; QC: quality control; QqQ: triple quadrupole; *r*^2^: determination factor; RSD: relative standard deviation; SPE: solid-phase extraction; UHPLC: ultra-high-performance liquid-chromatography; UPW: ultrapure water; UV: ultraviolet; UWWTD: urban wastewater treatment directive; WFD: water framework directive; WL: watch list; WWTP: wastewater treatment plant.
