# Supplementary material for: Multi-Residue Determination and Risk Assessment of EU-Relevant Pharmaceuticals, Pesticides, and UV-Filters in Drinking Water
Source: Pharmaceuticals (Basel). 2026 Feb 28;19(3):402. doi: 10.3390/ph19030402 (PMC13028605; doi:10.3390/ph19030402)
Supplement: Supplementary file 1 [file pharmaceuticals-19-00402-s001.zip › pharmaceuticals-4153975-supplementary.pdf]

Supplementary Material

Multi-Residue Determination and Risk Assessment of EU-Relevant Pharmaceuticals, Pesticides and UV-Filters in Drinking Water

Inês M. Quintela, Ana M. Gorito*, Marta O. Barbosa, Adrián M.T. Silva, Ana R.L. Ribeiro

LSRE-LCM, ALiCE, Faculty of Engineering, University of Porto, Rua Dr. Roberto Frias, 4200-465 Porto, Portugal

*Corresponding author: amgorito@fe.up.pt (A.M. Gorito)

Table of Contents

Table S1. Target compounds selected from the EU Watch List 2022 (*) [1] and 2025 (**) [2], and from the revised Urban Wastewater Treatment Directive (***) (Directive 2024/3019/EU) [3]. 3

Table S2. Multiple reaction monitoring (MRM) instrument parameters for tandem mass-spectrometry analysis of each target analyte selected from Watch List 2022 (*) [1] and 2025 (**) [2], and from the revised Urban Wastewater Treatment Directive (***) (Directive 2024/3019/EU) [3]. 13

Table S3. Retention time, range, linearity, instrument and method detection and quantification limits (IDL, IQL, MDL, and MQL) for the 23 target MPs from Watch List 2022 (*) [1] and 2025 (**) [2], and from the revised Urban Wastewater Treatment Directive (***) (Directive 2024/3019/EU) [3]. 16

Table S4. Absolute recovery, extraction efficiency, matrix effect, accuracy, and intra- and inter-batch precision for the 23 target MPs from Watch List 2022 (*) [1] and 2025 (**) [2], and from the revised Urban Wastewater Treatment Directive (***) (Directive 2024/3019/EU) [3]. 18

Table S5. Concentration in each sample (numbered from 1 to 50) of all 12 target pharmaceuticals. Quantifiable concentrations, i.e., those above the MQL, are highlighted in green and those below the MQL in yellow along with the corresponding MQL value. (n.d.: "not detected"). 20

Table S6. Concentration in each sample (numbered from 1 to 50) of all 9 target pesticides and 2 target UV-filters. Quantifiable concentrations, i.e., those above the MQL, are highlighted in green,

those bellow the MQL in yellow along with the corresponding MQL value, and those above the upper limit of its calibration curve in red along with its value. (n.d.: “not detected”). 24

Figure S1 MA whiteness assessment score of the SPE-LC-MS/MS method developed to determine 23 MPs in DW 28

Table S1. *Target compounds selected from the EU Watch List 2022 (*) [1] and 2025 (**) [2], and from the revised Urban Wastewater Treatment Directive (***) (Directive 2024/3019/EU) [3].*

**Class**	**Subclass**	**Compound name**	**CAS number**	**Chemical structure**	**Molecular formula[a]**	**$M_w^{a,b}$ ($g\ mol^{-1}$)**	**$pK_a^{a,b}$**
**Pesticide**	Fungicide	Azoxystrobin*	131860-33-8	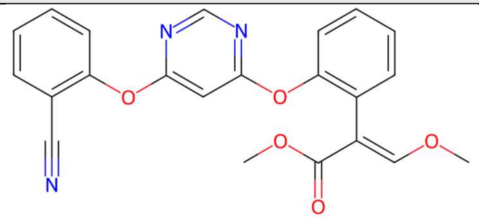	$C_{22}H_{17}N_3O_5$	403.394	1.94
		Dimoxystrobin*	149961-52-4	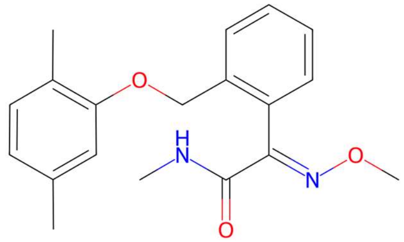	$C_{19}H_{22}N_2O_3$	326.396	11.29
		Imazalil*	35554-44-0	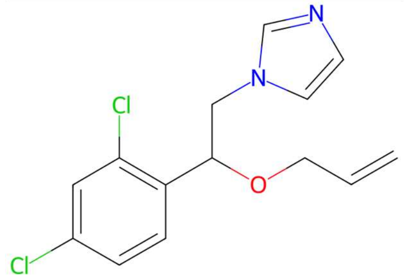	$C_{14}H_{14}Cl_2N_2O$	297.179	6.53

Table S1. Target compounds selected from the EU Watch List 2022 (*) [1] and 2025 (**) [2], and from the revised Urban Wastewater Treatment Directive (***) (Directive 2024/3019/EU) [3].

Class	Subclass	Compound name	CAS number	Chemical structure	Molecular formula[a]	M_w[a,b] (g mol^{-1})	pK_a[a,b]
**Pesticide**	Fungicide	Ipconazole*	125225-28-7	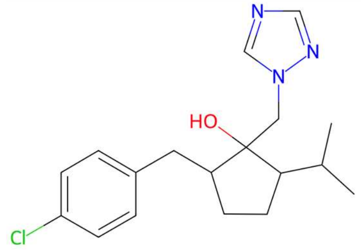	$C_{18}H_{24}ClN_3O$	333.860	13.76
		Metconazole*	125116-23-6	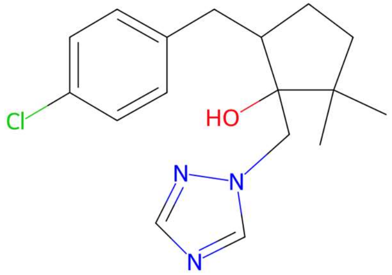	$C_{17}H_{22}ClN_3O$	319.830	13.82
		Penconazole*	66246-88-6	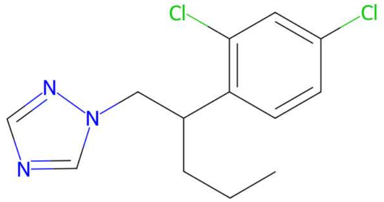	$C_{13}H_{15}Cl_2N_3$	284.184	2.80

Table S1. *Target compounds selected from the EU Watch List 2022 (*) [1] and 2025 (**) [2], and from the revised Urban Wastewater Treatment Directive (***) (Directive 2024/3019/EU) [3].*

**Class**	**Subclass**	**Compound name**	**CAS number**	**Chemical structure**	**Molecular formula[a]**	**M_w[a,b] (g mol^{-1})**	**pK_a[a,b]**
**Pesticide**	Fungicide	Prochloraz*	67747-09-5	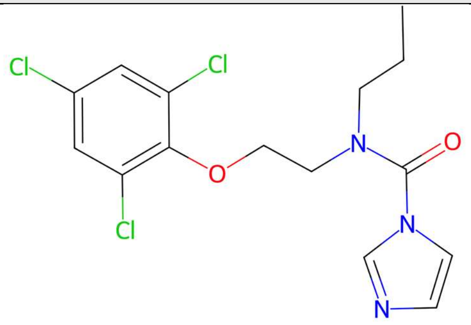	$C_{15}H_{16}Cl_3N_3O_2$	376.662	3.80
		Tetraconazole*	112281-77-3	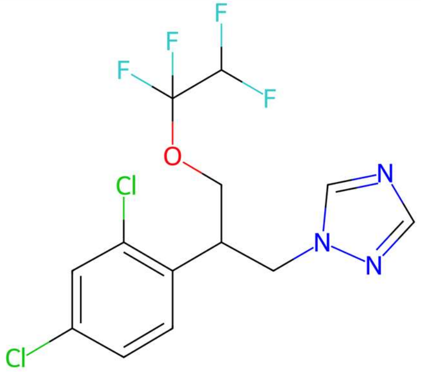	$C_{13}H_{11}Cl_2F_4N_3O$	372.143	2.68

Table S1. *Target compounds selected from the EU Watch List 2022 (*) [1] and 2025 (**) [2], and from the revised Urban Wastewater Treatment Directive (***) (Directive 2024/3019/EU) [3].*

Class	Subclass	Compound name	CAS number	Chemical structure	Molecular formula[a]	$M_w^{a,b}$ (g mol^{-1})	p$K_a^{a,b}$
**Pesticide**	Insecticide	Fipronil*,**	120068-37-3	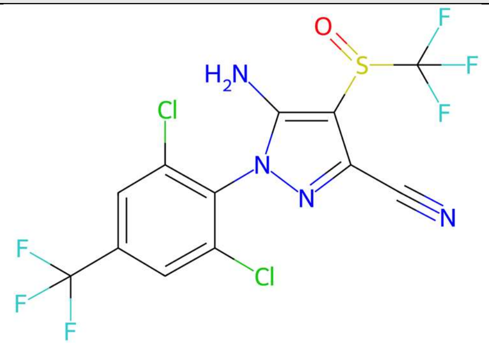	$C_{12}H_4Cl_2F_6N_4OS$	437.139	0.74
**Pharmaceutical**	Antibiotic	Clarithromycin***	81103-11-9	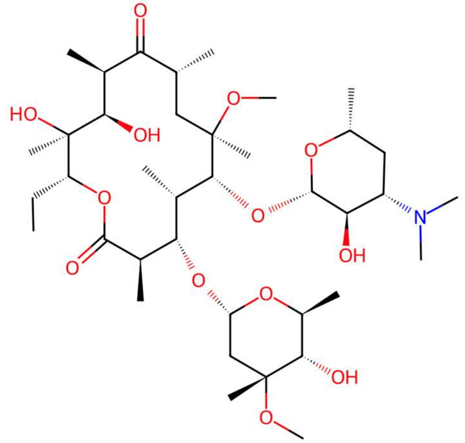	$C_{38}H_{69}NO_{13}$	747.964	8.99

Table S1. *Target compounds selected from the EU Watch List 2022 (*) [1] and 2025 (**) [2], and from the revised Urban Wastewater Treatment Directive (***) (Directive 2024/3019/EU) [3].*

**Class**	**Subclass**	**Compound name**	**CAS number**	**Chemical structure**	**Molecular formula**[a]	**M_w**[a,b] **(g mol^{-1})**	**pK_a**[a,b]
**Pharmaceutical**	Antibiotic	Clindamycin*,**	18323-44-9	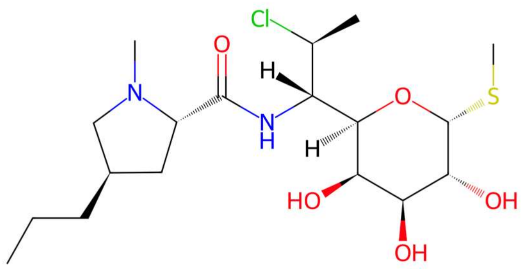	$C_{18}H_{33}ClN_2O_5S$	424.981	7.60
	Anticonvulsant	Carbamazepine***	298-46-4	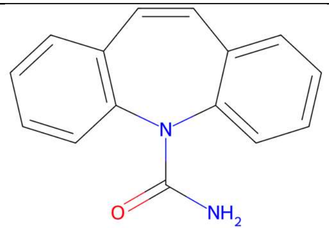	$C_{15}H_{12}N_2O$	236.274	15.96

__Table S1.__ Target compounds selected from the EU Watch List 2022 (*) [1] and 2025 (**) [2], and from the revised Urban Wastewater Treatment Directive (***) (Directive 2024/3019/EU) [3].

Class	Subclass	Compound name	CAS number	Chemical structure	Molecular formula[a]	M_w[a,b] (g mol^{-1})	pK_a[a,b]
**Pharmaceutical**	Antidepressant	Citalopram***	59729-33-8	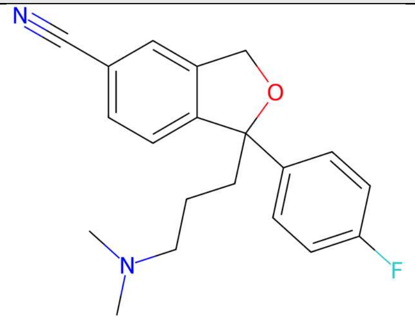	$C_{20}H_{21}FN_2O$	324.399	9.38
		O-desmethyl venlafaxine*	93413-62-8	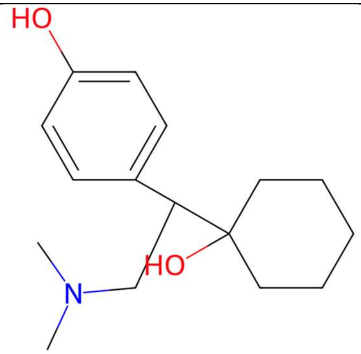	$C_{16}H_{25}NO_2$	263.381	10.11

Table S1. *Target compounds selected from the EU Watch List 2022 (*) [1] and 2025 (**) [2], and from the revised Urban Wastewater Treatment Directive (***) (Directive 2024/3019/EU) [3].*

**Class**	**Subclass**	**Compound name**	**CAS number**	**Chemical structure**	**Molecular formula**[a]	**M_w**[a,b] **(g mol^{-1})**	**pK_a**[a,b]
**Pharmaceutical**	Antihypertensive	Candesartan***	145040-37-5	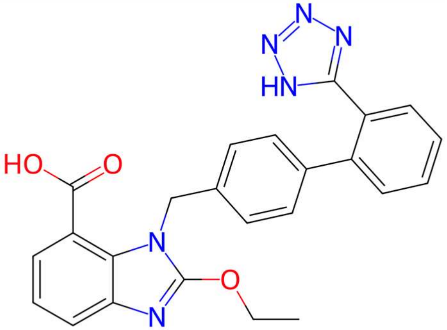	$C_{24}H_{20}N_6O_3$	440.463	4.23
		Irbesartan***	138402-11-6	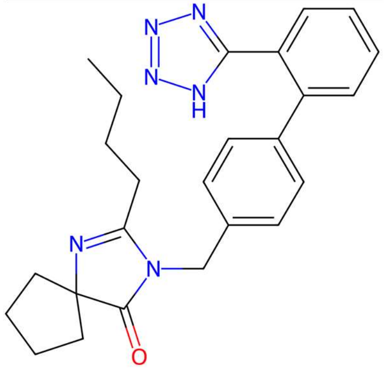	$C_{25}H_{28}N_6O$	428.540	4.08

Table S1. *Target compounds selected from the EU Watch List 2022 (*) [1] and 2025 (**) [2], and from the revised Urban Wastewater Treatment Directive (***) (Directive 2024/3019/EU) [3].*

Class	Subclass	Compound name	CAS number	Chemical structure	Molecular formula[a]	M_w[a,b] (g mol^{-1})	pK_a[a,b]
Pharmaceutical	Antipsychotic	Amisulpride***	71675-85-9	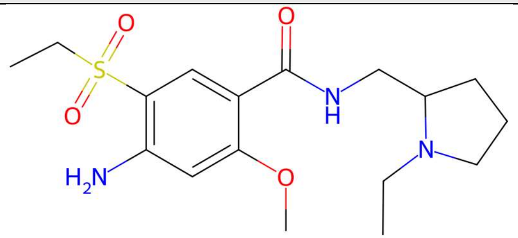	$C_{17}H_{27}N_3O_4S$	369.480	9.37
	Fungicide	Climbazole**	38083-17-9	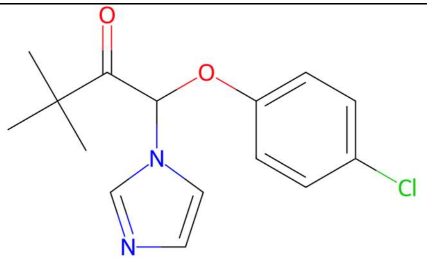	$C_{15}H_{17}ClN_2O_2$	292.763	5.66
		Clotrimazole*	23593-75-1	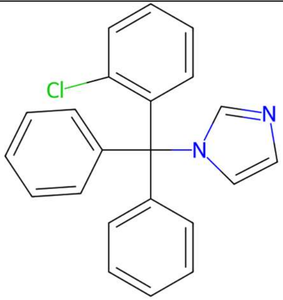	$C_{22}H_{17}ClN_2$	344.842	4.10

Table S1. *Target compounds selected from the EU Watch List 2022 (*) [1] and 2025 (**) [2], and from the revised Urban Wastewater Treatment Directive (***) (Directive 2024/3019/EU) [3].*

Class	Subclass	Compound name	CAS number	Chemical structure	Molecular formula[a]	M_w[a,b] (g mol^{-1})	pK_a[a,b]
**Pharmaceutical**	Fungicide	Fluconazole*	86386-73-4	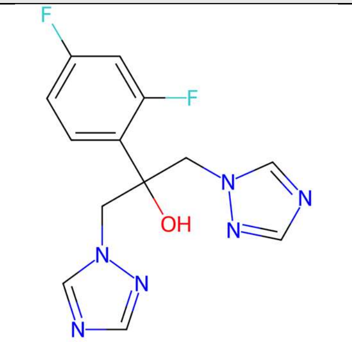	$C_{13}H_{12}F_2N_6O$	206.276	1.76
		Miconazole*	22916-47-8	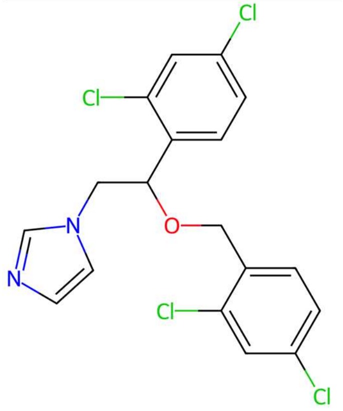	$C_{18}H_{14}Cl_4N_2O$	416.123	6.91

Table S1. *Target compounds selected from the EU Watch List 2022 (*) [1] and 2025 (**) [2], and from the revised Urban Wastewater Treatment Directive (***) (Directive 2024/3019/EU) [3].*

**Class**	**Subclass**	**Compound name**	**CAS number**	**Chemical structure**	**Molecular formula**[a]	**M_w**[a,b] **($g\ mol^{-1}$)**	**pK_a**[a,b]
**Organic UV-filter**	-	Benzotriazole***	95-14-7	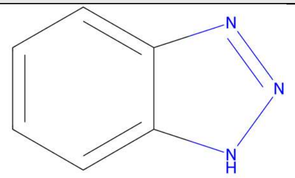	$C_6H_5N_3$	119.127	8.37
		Octocrylene*,**	6197-30-4	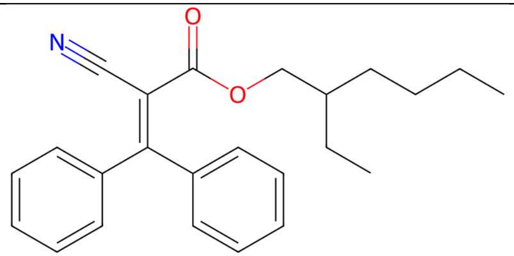	$C_{24}H_{27}NO_2$	361.485	n.a.

n.a.: not available; [a]PubChem; [b]DrugBank

[1] Commission Implementing Decision (EU) 2022/1307 of 22 July 2022 Establishing a Watch List of Substances for Union-Wide Monitoring in the Field of Water Policy Pursuant to Directive 2008/105/EC [Online]. 2022. Available online: http://data.europa.eu/eli/dec_impl/2022/1307/oj (accessed on 20 June 2025).
[2] Commission Implementing Decision (EU) 2025/439 of 28 February 2025 Establishing a Watch List of Substances for Union-Wide Monitoring in the Field of Water Policy Pursuant to Directive 2008/105/EC [Online]. 2025. Available online: https://eur-lex.europa.eu/eli/dec_impl/2025/439/oj (accessed on 20 June 2025).
[3] Directive (EU) 2024/3019 of 27 November 2024 Concerning Urban Wastewater Treatment (Recast) [Online]. 2024. Available online: https://eur-lex.europa.eu/eli/dir/2024/3019/oj/eng (accessed on 20 June 2025).

Table S2. *Multiple reaction monitoring (MRM) instrument parameters for tandem mass-spectrometry analysis of each target analyte selected from Watch List 2022 (*) [1] and 2025 (**) [2], and from the revised Urban Wastewater Treatment Directive (***) (Directive 2024/3019/EU) [3].*

Class (subclass)	Compound name	IS	ESI mode		Precursor ion (m/z)	Quantification (MRM1)				Qualification (MRM2)			
			PI/ NI	Molecular ion		Product ion (m/z)	DP (V)	CE (V)	CXP (V)	Product ion (m/z)	DP (V)	CE (V)	CX (V)
**Pesticide (Fungicide)**	Azoxystrobin*	(e)	PI	$[M+H]^+$	404.05	372.00	-19	-16	-25	344.00	-19	-25	-23
	Dimoxystrobin*	(e)	PI	$[M+H]^+$	327.15	205.00	-15	-11	-20	116.05	-15	-21	-20
	Imazalil*	(e)	NI	$[M-H]^-$	296.90	41.10	-30	-36	-13	158.90	-30	-23	-27
	Ipconazole*	(e)	PI	$[M+H]^+$	334.10	70.05	-16	-21	-27	124.95	-16	-43	-23
	Metconazole*	(e)	PI	$[M+H]^+$	320.10	70.05	-15	-25	-26	124.90	-15	-45	-21
	Penconazole*	(e)	PI	$[M+H]^+$	284.00	70.05	-28	-19	-26	158.95	-28	-25	-30
	Prochloraz*	(e)	PI	$[M+H]^+$	378.00	309.90	-18	-13	-30	70.05	-18	-26	-24
	Tetraconazole*	(e)	NI	$[M-H]^-$	371.90	158.90	-17	-35	-29	70.05	-17	-24	-25
**Pesticide (Insecticide)**	Fipronil*,**	(e)	NI	$[M-H]^-$	434.85	329.95	15	16	23	250.00	15	28	25
**Pharmaceutical (Antibiotic)**	Clarithromycin***	(d)	PI	$[M+H]^+$	748.40	158.15	-40	-30	-15	590.30	-40	-21	-28
	Clindamycin*,**	(b)	PI	$[M+H]^+$	425.00	126.15	-20	-30	-23	377.10	-20	-21	-25

Table S2. *Multiple reaction monitoring (MRM) instrument parameters for tandem mass-spectrometry analysis of each target analyte selected from Watch List 2022 (*) [1] and 2025 (**) [2], and from the revised Urban Wastewater Treatment Directive (***) (Directive 2024/3019/EU) [3].*

Class (subclass)	Compound name	IS	ESI mode		Precursor ion (m/z)	Quantification (MRM1)				Qualification (MRM2)			
			PI/ NI	Molecular ion		Product ion (m/z)	DP (V)	CE (V)	CXP (V)	Product ion (m/z)	DP (V)	CE (V)	CX (V)
**Pharmaceutical (Anticonvulsant)**	Carbamazepine***	(a)	PI	$[M+H]^-$	236.90	194.05	-19	-19	-18	192.00	-19	-22	-18
**Pharmaceutical (Antidepressant)**	Citalopram***	(c)	PI	$[M+H]^+$	324.80	109.00	-30	-28.0	-20	262.10	-30	-19	-27
	O-desmethyl venlafaxine*	(f)	PI	$[M+H]^+$	264.10	58.05	-27	-21.0	-22	246.15	-27	-13	-26
	Candesartan***	(d)	PI	$[M+H]^+$	441.20	263.20	-21	-13.0	-27	423.15	-21	-11	-19
	Irbesartan***	(e)	PI	$[M+H]^+$	429.20	207.00	-21	-26.0	-21	195.10	-21	-24	-12
**Pharmaceutical (Antipsychotic)**	Amisulpride***	(e)	PI	$[M+H]^+$	370.10	242.05	-17	-29.0	-15	196.00	-17	-42	-18
**Pharmaceutical (Fungicide)**	Climbazole**	(e)	PI	$[M+H]^+$	293.05	69.05	-14	-23	-27	197.10	-14	-16	-20
	Clotrimazole*	(e)	PI	$[M+H]^+$	345.05	277.10	-17	-9	-28	165.00	-17	-33	-30
	Fluconazole*	(e)	PI	$[M+H]^+$	307.05	220.10	-15	-21	-22	238.10	-15	-17	-24
	Miconazole*	(e)	PI	$[M+H]^+$	416.95	161.00	-20	-45	-27	159.00	-20	-46	-27

Table S2. *Multiple reaction monitoring (MRM) instrument parameters for tandem mass-spectrometry analysis of each target analyte selected from Watch List 2022 (*) [1] and 2025 (**) [2], and from the revised Urban Wastewater Treatment Directive (***) (Directive 2024/3019/EU) [3].*

Class (subclass)	Compound name	IS	ESI mode		Precursor ion (m/z)	Quantification (MRM1)				Qualification (MRM2)			
			PI/ NI	Molecular ion		Product ion (m/z)	DP (V)	CE (V)	CXP (V)	Product ion (m/z)	DP (V)	CE (V)	CX (V)
**UV-filter** (-)	Benzotriazole***	(a)	PI	$[M+H]^+$	120.00	65.05	-12	-25	-22	38.95	-12	-47	-30
	Octocrylene*,**	(a)	PI	$[M+Na]^+$	379.00	250.05	-27	-13	-11	232.10	-27	-25	-24
**Internal Standards**	Carbamazepine-d_{10} (a)	-	PI	$[M+H]^+$	247.15	204.15	-26	-20	-12	-	-	-	-
	Ciprofloxacin-d_8 (b)	-	PI	$[M+H]^+$	340.00	322.20	-27	-22	-30	-	-	-	-
	Citalopram-d_6 (c)	-	PI	$[M+H]^+$	330.80	109.10	-28	-28	-19	-	-	-	-
	Ofloxacin-d_3 (d)	-	PI	$[M+H]^+$	364.90	321.15	-27	-21	-21	-	-	-	-
	Prochloraz-d_4 (e)	-	PI	$[M+H]^+$	382.20	313.95	-20	-13	-22	-	-	-	-
	Venlafaxine-d_6 (f)	-	PI	$[M+H]^+$	283.90	64.20	-23	-21	-23	-	-	-	-

CE: collision energy; CXP: collision cell exit potential; DP: decluttering potential; IS: internal standard; NI: negative ionisation mode; PI: positive ionisation mode.

[1] Commission Implementing Decision (EU) 2022/1307 of 22 July 2022 Establishing a Watch List of Substances for Union-Wide Monitoring in the Field of Water Policy Pursuant to Directive 2008/105/EC [Online]. 2022. Available online: http://data.europa.eu/eli/dec_impl/2022/1307/oj (accessed on 20 June 2025).

[2] Commission Implementing Decision (EU) 2025/439 of 28 February 2025 Establishing a Watch List of Substances for Union-Wide Monitoring in the Field of Water Policy Pursuant to Directive 2008/105/EC [Online]. 2025. Available online: https://eur-lex.europa.eu/eli/dec_impl/2025/439/oj (accessed on 20 June 2025).

[3] Directive (EU) 2024/3019 of 27 November 2024 Concerning Urban Wastewater Treatment (Recast) [Online]. 2024. Available online: https://eur-lex.europa.eu/eli/dir/2024/3019/oj/eng (accessed on 20 June 2025).

Table S3. *Retention time, range, linearity, instrument and method detection and quantification limits (IDL, IQL, MDL, and MQL) for the 23 target MPs from Watch List 2022 (*) [1] and 2025 (**) [2], and from the revised Urban Wastewater Treatment Directive (***) (Directive 2024/3019/EU) [3].*

Class	Analyte	Retention time (min)	Range (ng L^{-1})	r^2	IDL (µg L^{-1})	IQL (µg L^{-1})	MDL (ng L^{-1})	MQL (µg L^{-1})
Pharmaceutical	Amisulpride***	12.4	5.00 – 133	0.995	0.44	1.33	0.06	0.19
	Candesartan***	20.9	5.00 – 133	0.999	1.53	4.64	0.31	0.95
	Carbamazepine***	16.5	5.00 – 133	0.997	0.39	1.17	0.05	0.16
	Citalopram***	18.4	5.00 – 117	0.997	2.69	8.18	0.12	0.37
	Clarithromycin***	23.3	5.00 – 133	0.996	0.33	0.99	0.06	0.19
	Climbazole**	20.2	10.0 – 117	0.996	0.36	1.08	0.14	0.41
	Clindamycin*,**	15.5	5.00 – 133	0.996	1.78	5.38	0.29	0.88
	Clotrimazole*	21.9	10.0 – 117	0.998	0.24	0.74	0.11	0.35
	Fluconazole*	12.1	5.00 – 133	0.996	0.07	0.20	0.03	0.09
	Irbesartan***	20.6	5.00 – 133	0.997	0.10	0.30	0.04	0.13
	Miconazole*	25.4	5.00 – 100	0.999	0.30	0.91	0.12	0.35
	O-desmethylvenlafaxine*	10.4	5.00 – 133	0.996	0.05	0.17	0.02	0.08
Pesticide	Azoxystrobin*	21.2	5.00 – 133	0.997	1.80	5.47	0.70	2.13
	Dimoxystrobin*	23.0	5.00 – 117	0.999	0.30	0.92	0.20	0.61

Table S3. *Retention time, range, linearity, instrument and method detection and quantification limits (IDL, IQL, MDL, and MQL) for the 23 target MPs from Watch List 2022 (*) [1] and 2025 (**) [2], and from the revised Urban Wastewater Treatment Directive (***) (Directive 2024/3019/EU) [3].*

Class	Analyte	Retention time (min)	Range (ng L^{-1})	r^2	IDL (µg L^{-1})	IQL (µg L^{-1})	MDL (ng L^{-1})	MQL (µg L^{-1})
**Pesticide**	Imazalil*	20.0	5.00 – 100	0.996	0.60	1.83	0.18	0.55
	Fipronil*,**	24.1	5.00 – 133	0.996	0.11	0.33	0.06	0.18
	Ipconazole*	24.0	5.00 – 133	0.995	0.20	0.62	0.11	0.32
	Metconazole*	23.1	10.0 – 133	0.996	0.11	0.34	0.50	0.15
	Penconazole*	22.3	5.00 – 133	0.998	5.69	17.2	1.47	4.45
	Prochloraz*	23.5	40.0 – 133	0.997	0.88	2.66	0.12	0.38
	Tetraconazole*	22.3	5.00 – 133	0.999	0.61	1.84	0.25	0.75
**UV-filter**	Benzotriazole***	8.5	5.00 – 133	0.996	0.12	0.36	0.06	0.19
	Octocrylene*,**	27.4	5.00 – 133	0.995	0.29	0.87	0.16	0.48

[1] Commission Implementing Decision (EU) 2022/1307 of 22 July 2022 Establishing a Watch List of Substances for Union-Wide Monitoring in the Field of Water Policy Pursuant to Directive 2008/105/EC [Online]. 2022. Available online: http://data.europa.eu/eli/dec_impl/2022/1307/oj (accessed on 20 June 2025).
[2] Commission Implementing Decision (EU) 2025/439 of 28 February 2025 Establishing a Watch List of Substances for Union-Wide Monitoring in the Field of Water Policy Pursuant to Directive 2008/105/EC [Online]. 2025. Available online: https://eur-lex.europa.eu/eli/dec_impl/2025/439/oj (accessed on 20 June 2025).
[3] Directive (EU) 2024/3019 of 27 November 2024 Concerning Urban Wastewater Treatment (Recast) [Online]. 2024. Available online: https://eur-lex.europa.eu/eli/dir/2024/3019/oj/eng (accessed on 20 June 2025).

Table S4. *Absolute recovery, extraction efficiency, matrix effect, accuracy, and intra- and inter-batch precision for the 23 target MPs from Watch List 2022 (*) [1] and 2025 (**) [2], and from the revised Urban Wastewater Treatment Directive (***) (Directive 2024/3019/EU) [3].*

Class	Analyte	Absolute recovery (%)	Extraction efficiency (%)	Matrix effect (%)	Accuracy (%)	Intra-batch precision (%)	Inter-batch precision (%)
Pharmaceutical	Amisulpride***	21.9	5.46	16.4	102 ± 17.0	9.57 – 18.1	6.55
	Candesartan***	30.8	62.4	-31.6	85.5 ± 13.5	16.6 – 16.8	6.77
	Carbamazepine***	20.6	54.1	-33.5	94.5 ± 8.94	2.89 – 4.56	2.56
	Citalopram***	6.74	0.80	5.94	116 ± 6.52	7.82 – 11.1	5.77
	Clarithromycin***	29.0	1.89	27.1	108 ± 8.45	4.75 – 13.7	7.85
	Climbazole**	57.0	29.6	27.4	111 ± 3.87	7.07 – 10.2	7.77
	Clindamycin*,**	24.5	7.49	17.0	99.7 ± 11.6	9.96 – 11.1	7.41
	Clotrimazole*	69.9	33.0	37.0	112 ± 2.87	8.41 – 13.9	6.68
	Fluconazole*	71.4	27.8	43.6	110 ± 17.0	5.89 – 15.5	8.38
	Irbesartan***	63.2	95.5	-32.3	105 ± 14.1	7.36 – 11.5	8.04
	Miconazole*	58.1	10.2	47.9	119 ± 2.31	2.44 – 10.1	5.55
	O-desmethylvenlafaxine*	66.5	13.6	52.9	120 ± 11.4	4.31 – 8.26	3.58
Pesticide	Azoxystrobin*	58.4	43.0	15.4	106 ± 3.12	6.81 – 12.3	5.55
	Dimoxystrobin*	99.1	30.5	68.6	105 ± 4.66	5.51 – 11.1	2.45

Table S4. *Absolute recovery, extraction efficiency, matrix effect, accuracy, and intra- and inter-batch precision for the 23 target MPs from Watch List 2022 (*) [1] and 2025 (**) [2], and from the revised Urban Wastewater Treatment Directive (***) (Directive 2024/3019/EU) [3].*

Class	Analyte	Absolute recovery (%)	Extraction efficiency (%)	Matrix effect (%)	Accuracy (%)	Intra-batch precision (%)	Inter-batch precision (%)
**Pesticide**	Imazalil*	45.6	36.4	9.13	119 ± 14.3	5.87 – 9.79	7.28
	Fipronil*,**	79.6	40.4	39.2	114 ± 13.7	12.2 – 12.8	6.11
	Ipconazole*	78.1	55.7	22.4	98.5 ± 11.7	11.4 – 14.2	7.55
	Metconazole*	66.5	26.9	39.7	105 ± 5.79	5.81 – 15.7	4.98
	Penconazole*	38.7	26.7	12.0	101 ± 5.13	5.81 – 12.3	5.77
	Prochloraz*	21.2	41.9	-20.7	88.5 ± 5.15	4.60 – 8.94	10.4
	Tetraconazole*	61.5	53.0	8.50	104 ± 9.65	0.92 – 11.5	5.73
**UV-filter**	Benzotriazole***	77.2	14.1	63.1	114 ± 18.2	12.2 – 13.9	7.66
	Octocrylene*,**	82.8	25.9	56.9	92.0 ± 0.23	7.87 – 11.3	1.21

[1] Commission Implementing Decision (EU) 2022/1307 of 22 July 2022 Establishing a Watch List of Substances for Union-Wide Monitoring in the Field of Water Policy Pursuant to Directive 2008/105/EC [Online]. 2022. Available online: http://data.europa.eu/eli/dec_impl/2022/1307/oj (accessed on 20 June 2025).

[2] Commission Implementing Decision (EU) 2025/439 of 28 February 2025 Establishing a Watch List of Substances for Union-Wide Monitoring in the Field of Water Policy Pursuant to Directive 2008/105/EC [Online]. 2025. Available online: https://eur-lex.europa.eu/eli/dec_impl/2025/439/oj (accessed on 20 June 2025).

[3] Directive (EU) 2024/3019 of 27 November 2024 Concerning Urban Wastewater Treatment (Recast) [Online]. 2024. Available online: https://eur-lex.europa.eu/eli/dir/2024/3019/oj/eng (accessed on 20 June 2025).

Table S5. *Concentration in each sample (numbered from 1 to 50) of all 12 target pharmaceuticals. Quantifiable concentrations, i.e., those above the MQL, are highlighted in green and those below the MQL in yellow along with the corresponding MQL value. (n.d.: "not detected").*

(ng L^{-1})	AMS	CDS	CBZ	CTL	CLR	CLZ	CLN	CTZ	FLZ	IRB	MCZ	ODV
**1**	n.d.	n.d.	n.d.	n.d.	n.d.	n.d.	n.d.	n.d.	n.d.	n.d.	n.d.	n.d.
**2**	n.d.	n.d.	n.d.	n.d.	n.d.	n.d.	n.d.	n.d.	n.d.	n.d.	n.d.	n.d.
**3**	n.d.	n.d.	n.d.	n.d.	n.d.	n.d.	n.d.	n.d.	n.d.	n.d.	n.d.	n.d.
**4**	n.d.	n.d.	n.d.	n.d.	n.d.	n.d.	n.d.	n.d.	n.d.	n.d.	n.d.	n.d.
**5**	n.d.	n.d.	n.d.	n.d.	n.d.	n.d.	n.d.	n.d.	n.d.	n.d.	n.d.	n.d.
**6**	n.d.	n.d.	n.d.	n.d.	n.d.	n.d.	n.d.	n.d.	n.d.	n.d.	n.d.	n.d.
**7**	n.d.	n.d.	n.d.	n.d.	n.d.	n.d.	n.d.	n.d.	n.d.	n.d.	n.d.	n.d.
**8**	n.d.	n.d.	n.d.	n.d.	n.d.	n.d.	n.d.	n.d.	n.d.	0.37±2.22	n.d.	n.d.
**9**	n.d.	n.d.	n.d.	n.d.	n.d.	n.d.	n.d.	n.d.	n.d.	n.d.	n.d.	n.d.
**10**	n.d.	n.d.	n.d.	n.d.	n.d.	n.d.	n.d.	n.d.	n.d.	n.d.	n.d.	n.d.
**11**	n.d.	n.d.	n.d.	n.d.	n.d.	n.d.	n.d.	n.d.	n.d.	n.d.	n.d.	n.d.
**12**	n.d.	n.d.	n.d.	n.d.	n.d.	n.d.	n.d.	n.d.	3.88±3.06	n.d.	n.d.	n.d.
**13**	n.d.	n.d.	n.d.	n.d.	n.d.	n.d.	n.d.	n.d.	n.d.	n.d.	n.d.	n.d.
**14**	n.d.	n.d.	n.d.	n.d.	n.d.	n.d.	n.d.	n.d.	n.d.	0.28±2.22	n.d.	n.d.

Table S5. *Concentration in each sample (numbered from 1 to 50) of all 12 target pharmaceuticals. Quantifiable concentrations, i.e., those above the MQL, are highlighted in green and those below the MQL in yellow along with the corresponding MQL value. (n.d.: "not detected").*

(ng L^{-1})	**AMS**	**CDS**	**CBZ**	**CTL**	**CLR**	**CLZ**	**CLN**	**CTZ**	**FLZ**	**IRB**	**MCZ**	**ODV**
**15**	n.d.	n.d.	n.d.	n.d.	n.d.	n.d.	n.d.	n.d.	38.4±2.66	2.43±2.19	n.d.	n.d.
**16**	n.d.	n.d.	n.d.	n.d.	n.d.	n.d.	n.d.	n.d.	n.d.	n.d.	n.d.	n.d.
**17**	n.d.	n.d.	n.d.	n.d.	n.d.	n.d.	n.d.	n.d.	n.d.	n.d.	n.d.	n.d.
**18**	n.d.	n.d.	n.d.	n.d.	n.d.	n.d.	n.d.	n.d.	n.d.	n.d.	n.d.	n.d.
**19**	n.d.	n.d.	n.d.	n.d.	n.d.	n.d.	n.d.	n.d.	n.d.	n.d.	n.d.	n.d.
**20**	n.d.	n.d.	n.d.	n.d.	n.d.	n.d.	n.d.	n.d.	n.d.	n.d.	n.d.	n.d.
**21**	n.d.	n.d.	n.d.	n.d.	n.d.	n.d.	n.d.	n.d.	n.d.	n.d.	n.d.	n.d.
**22**	n.d.	n.d.	n.d.	n.d.	n.d.	n.d.	n.d.	n.d.	n.d.	n.d.	n.d.	n.d.
**23**	n.d.	n.d.	n.d.	n.d.	n.d.	n.d.	n.d.	n.d.	n.d.	n.d.	n.d.	n.d.
**24**	n.d.	n.d.	n.d.	n.d.	n.d.	n.d.	n.d.	n.d.	n.d.	n.d.	n.d.	n.d.
**25**	n.d.	n.d.	n.d.	n.d.	n.d.	n.d.	n.d.	n.d.	n.d.	n.d.	n.d.	n.d.
**26**	n.d.	n.d.	n.d.	n.d.	n.d.	n.d.	n.d.	n.d.	n.d.	n.d.	n.d.	n.d.
**27**	n.d.	n.d.	n.d.	n.d.	n.d.	n.d.	n.d.	n.d.	n.d.	n.d.	n.d.	n.d.
**28**	n.d.	n.d.	n.d.	n.d.	n.d.	n.d.	n.d.	n.d.	n.d.	n.d.	n.d.	n.d.

Table S5. *Concentration in each sample (numbered from 1 to 50) of all 12 target pharmaceuticals. Quantifiable concentrations, i.e., those above the MQL, are highlighted in green and those below the MQL in yellow along with the corresponding MQL value. (n.d.: "not detected").*

(ng L^{-1})	**AMS**	**CDS**	**CBZ**	**CTL**	**CLR**	**CLZ**	**CLN**	**CTZ**	**FLZ**	**IRB**	**MCZ**	**ODV**
**29**	n.d.	n.d.	n.d.	n.d.	n.d.	n.d.	n.d.	n.d.	n.d.	n.d.	n.d.	n.d.
**30**	n.d.	n.d.	n.d.	n.d.	n.d.	n.d.	n.d.	n.d.	n.d.	n.d.	n.d.	n.d.
**31**	n.d.	n.d.	n.d.	n.d.	n.d.	n.d.	n.d.	n.d.	n.d.	n.d.	n.d.	n.d.
**32**	n.d.	n.d.	n.d.	n.d.	n.d.	n.d.	n.d.	n.d.	n.d.	n.d.	n.d.	n.d.
**33**	n.d.	n.d.	n.d.	n.d.	n.d.	n.d.	n.d.	n.d.	n.d.	n.d.	n.d.	n.d.
**34**	n.d.	n.d.	n.d.	n.d.	n.d.	n.d.	n.d.	n.d.	n.d.	n.d.	n.d.	n.d.
**35**	n.d.	n.d.	n.d.	n.d.	n.d.	n.d.	n.d.	n.d.	n.d.	n.d.	n.d.	n.d.
**36**	n.d.	n.d.	n.d.	n.d.	n.d.	n.d.	n.d.	n.d.	n.d.	n.d.	n.d.	n.d.
**37**	n.d.	n.d.	n.d.	n.d.	n.d.	n.d.	n.d.	n.d.	n.d.	n.d.	n.d.	n.d.
**38**	n.d.	n.d.	n.d.	n.d.	n.d.	n.d.	n.d.	n.d.	n.d.	n.d.	n.d.	n.d.
**39**	n.d.	n.d.	n.d.	n.d.	n.d.	n.d.	n.d.	n.d.	n.d.	n.d.	n.d.	n.d.
**40**	n.d.	n.d.	n.d.	n.d.	n.d.	n.d.	n.d.	n.d.	n.d.	n.d.	n.d.	n.d.
**41**	n.d.	n.d.	n.d.	n.d.	n.d.	n.d.	n.d.	n.d.	n.d.	n.d.	n.d.	n.d.

Table S5. *Concentration in each sample (numbered from 1 to 50) of all 12 target pharmaceuticals. Quantifiable concentrations, i.e., those above the MQL, are highlighted in green and those below the MQL in yellow along with the corresponding MQL value. (n.d.: "not detected").*

(ng L^{-1})	AMS	CDS	CBZ	CTL	CLR	CLZ	CLN	CTZ	FLZ	IRB	MCZ	ODV
**42**	n.d.	n.d.	n.d.	n.d.	n.d.	n.d.	n.d.	n.d.	n.d.	n.d.	n.d.	n.d.
**43**	n.d.	n.d.	n.d.	n.d.	n.d.	n.d.	n.d.	n.d.	n.d.	n.d.	n.d.	n.d.
**44**	n.d.	n.d.	n.d.	n.d.	n.d.	n.d.	n.d.	n.d.	n.d.	n.d.	n.d.	n.d.
**45**	n.d.	n.d.	n.d.	n.d.	n.d.	n.d.	n.d.	n.d.	n.d.	n.d.	n.d.	n.d.
**46**	n.d.	n.d.	n.d.	n.d.	n.d.	n.d.	n.d.	n.d.	n.d.	n.d.	n.d.	n.d.
**47**	n.d.	n.d.	n.d.	n.d.	n.d.	n.d.	n.d.	n.d.	n.d.	n.d.	n.d.	n.d.
**48**	n.d.	n.d.	n.d.	n.d.	n.d.	n.d.	n.d.	n.d.	n.d.	n.d.	n.d.	n.d.
**49**	n.d.	n.d.	n.d.	n.d.	n.d.	n.d.	n.d.	n.d.	n.d.	n.d.	n.d.	n.d.
**50**	n.d.	n.d.	n.d.	n.d.	n.d.	n.d.	n.d.	n.d.	n.d.	n.d.	n.d.	n.d.

AMS: amisulpride; **CDS**: candesartan; **CBZ**: carbamazepine; **CTL**: citalopram; **CLR**: clarithromycin; **CLZ**: climbazole; **CLN**: clindamycin; **CTZ**: clotrimazole; **FLZ**: fluconazole; **IRB**: irbesartan; **MCZ**: miconazole; **ODV**: o-desmethylvenlafaxine.

Table S6. *Concentration in each sample (numbered from 1 to 50) of all 9 target pesticides and 2 target UV-filters. Quantifiable concentrations, i.e., those above the MQL, are highlighted in green, those bellow the MQL in yellow along with the corresponding MQL value, and those above the upper limit of its calibration curve in red along with its value. (n.d.: "not detected").*

(ng L^{-1})	AZO	DMX	IMZ	FIP	IPC	PEN	PCL	TRC	MTZ	BZT	OCT
**1**	n.d.	n.d.	n.d.	n.d.	n.d.	n.d.	n.d.	n.d.	n.d.	18.7±2.18	n.d.
**2**	n.d.	6.31±1.54	n.d.	n.d.	n.d.	n.d.	n.d.	n.d.	n.d.	5.65±2.32	n.d.
**3**	n.d.	n.d.	n.d.	n.d.	n.d.	n.d.	n.d.	n.d.	n.d.	n.d.	n.d.
**4**	n.d.	12.2±1.48	n.d.	n.d.	n.d.	n.d.	n.d.	n.d.	n.d.	1.23±2.36	n.d.
**5**	n.d.	n.d.	n.d.	n.d.	n.d.	n.d.	n.d.	n.d.	n.d.	16.7±2.19	n.d.
**6**	n.d.	n.d.	n.d.	n.d.	n.d.	n.d.	n.d.	n.d.	n.d.	23.2±2.14	n.d.
**7**	n.d.	n.d.	n.d.	n.d.	n.d.	n.d.	n.d.	n.d.	n.d.	36.2±2.05	n.d.
**8**	n.d.	37.1±1.31	n.d.	n.d.	n.d.	n.d.	n.d.	n.d.	n.d.	55.8±2.00	n.d.
**9**	n.d.	n.d.	n.d.	n.d.	n.d.	n.d.	n.d.	n.d.	n.d.	n.d.	n.d.
**10**	n.d.	n.d.	n.d.	n.d.	n.d.	n.d.	n.d.	n.d.	n.d.	30.4±2.09	n.d.
**11**	n.d.	< 0.61	n.d.	n.d.	n.d.	n.d.	n.d.	n.d.	n.d.	7.84±2.28	n.d.
**12**	n.d.	n.d.	n.d.	n.d.	n.d.	n.d.	n.d.	n.d.	n.d.	40.2±2.04	n.d.
**13**	n.d.	n.d.	n.d.	n.d.	n.d.	n.d.	n.d.	n.d.	n.d.	15.9±2.20	n.d.
**14**	n.d.	n.d.	n.d.	n.d.	n.d.	n.d.	n.d.	n.d.	n.d.	19.0±2.17	n.d.

Table S6. *Concentration in each sample (numbered from 1 to 50) of all 9 target pesticides and 2 target UV-filters. Quantifiable concentrations, i.e., those above the MQL, are highlighted in green, those bellow the MQL in yellow along with the corresponding MQL value, and those above the upper limit of its calibration curve in red along with its value. (n.d.: "not detected").*

(ng L^{-1})	AZO	DMX	IMZ	FIP	IPC	PEN	PCL	TRC	MTZ	BZT	OCT
**15**	n.d.	n.d.	n.d.	n.d.	n.d.	n.d.	n.d.	n.d.	n.d.	11.3±2.35	n.d.
**16**	n.d.	n.d.	n.d.	n.d.	n.d.	n.d.	n.d.	n.d.	n.d.	3.92±2.33	n.d.
**17**	n.d.	n.d.	n.d.	n.d.	n.d.	n.d.	n.d.	n.d.	n.d.	3.09±2.34	n.d.
**18**	n.d.	> 117	n.d.	n.d.	n.d.	n.d.	n.d.	n.d.	n.d.	3.07±2.34	n.d.
**19**	n.d.	< 0.61	n.d.	n.d.	n.d.	n.d.	n.d.	n.d.	n.d.	1.84±2.35	n.d.
**20**	n.d.	n.d.	n.d.	n.d.	n.d.	n.d.	n.d.	n.d.	n.d.	98.8±2.22	n.d.
**21**	n.d.	n.d.	n.d.	n.d.	n.d.	n.d.	n.d.	n.d.	n.d.	2.55±2.34	n.d.
**22**	n.d.	n.d.	n.d.	n.d.	n.d.	n.d.	n.d.	n.d.	n.d.	4.31±2.32	n.d.
**23**	n.d.	26.5±1.37	n.d.	n.d.	n.d.	n.d.	n.d.	n.d.	n.d.	12.7±2.23	n.d.
**24**	n.d.	n.d.	n.d.	n.d.	n.d.	n.d.	n.d.	n.d.	n.d.	n.d.	n.d.
**25**	n.d.	n.d.	n.d.	n.d.	n.d.	n.d.	n.d.	n.d.	n.d.	13.7±2.22	n.d.
**26**	n.d.	n.d.	n.d.	n.d.	n.d.	n.d.	n.d.	n.d.	n.d.	10.4±2.26	n.d.
**27**	n.d.	n.d.	n.d.	n.d.	n.d.	n.d.	n.d.	n.d.	n.d.	33.3±2.07	n.d.
**28**	n.d.	n.d.	n.d.	n.d.	n.d.	n.d.	n.d.	n.d.	n.d.	1.51±2.36	n.d.

Table S6. *Concentration in each sample (numbered from 1 to 50) of all 9 target pesticides and 2 target UV-filters. Quantifiable concentrations, i.e., those above the MQL, are highlighted in green, those bellow the MQL in yellow along with the corresponding MQL value, and those above the upper limit of its calibration curve in red along with its value. (n.d.: "not detected").*

(ng L^{-1})	AZO	DMX	IMZ	FIP	IPC	PEN	PCL	TRC	MTZ	BZT	OCT
**29**	n.d.	n.d.	n.d.	n.d.	n.d.	n.d.	n.d.	n.d.	n.d.	12.1±2.34	n.d.
**30**	n.d.	n.d.	n.d.	n.d.	n.d.	n.d.	n.d.	n.d.	n.d.	1.98±2.35	n.d.
**31**	n.d.	n.d.	n.d.	n.d.	n.d.	n.d.	n.d.	n.d.	n.d.	13.9±2.22	n.d.
**32**	n.d.	n.d.	n.d.	n.d.	n.d.	n.d.	n.d.	n.d.	n.d.	17.9±2.18	n.d.
**33**	n.d.	n.d.	n.d.	n.d.	n.d.	n.d.	n.d.	n.d.	n.d.	5.20±2.31	n.d.
**34**	n.d.	n.d.	n.d.	n.d.	n.d.	n.d.	n.d.	n.d.	n.d.	13.8±2.22	n.d.
**35**	n.d.	n.d.	n.d.	n.d.	n.d.	n.d.	n.d.	n.d.	n.d.	21.1±2.16	n.d.
**36**	n.d.	n.d.	n.d.	n.d.	n.d.	n.d.	n.d.	n.d.	n.d.	38.9±2.04	n.d.
**37**	n.d.	n.d.	n.d.	n.d.	n.d.	n.d.	n.d.	n.d.	n.d.	24.9±2.13	n.d.
**38**	n.d.	n.d.	n.d.	n.d.	n.d.	n.d.	n.d.	n.d.	n.d.	> 133	n.d.
**39**	n.d.	n.d.	n.d.	n.d.	n.d.	n.d.	n.d.	n.d.	n.d.	n.d.	n.d.
**40**	n.d.	n.d.	n.d.	n.d.	n.d.	n.d.	n.d.	n.d.	n.d.	1.86±2.35	n.d.
**41**	n.d.	n.d.	n.d.	n.d.	n.d.	n.d.	n.d.	n.d.	n.d.	n.d.	n.d.

Table S6. *Concentration in each sample (numbered from 1 to 50) of all 9 target pesticides and 2 target UV-filters. Quantifiable concentrations, i.e., those above the MQL, are highlighted in green, those bellow the MQL in yellow along with the corresponding MQL value, and those above the upper limit of its calibration curve in red along with its value. (n.d.: "not detected").*

(ng L^{-1})	**AZO**	**DMX**	**IMZ**	**FIP**	**IPC**	**PEN**	**PCL**	**TRC**	**MTZ**	**BZT**	**OCT**
**42**	n.d.	n.d.	n.d.	n.d.	n.d.	n.d.	n.d.	n.d.	n.d.	2.05±2.35	n.d.
**43**	n.d.	> 117	n.d.	n.d.	n.d.	n.d.	n.d.	n.d.	n.d.	1.19±2.36	n.d.
**44**	n.d.	n.d.	n.d.	n.d.	n.d.	n.d.	n.d.	n.d.	n.d.	n.d.	n.d.
**45**	n.d.	n.d.	n.d.	n.d.	n.d.	n.d.	n.d.	n.d.	n.d.	0.95±2.36	n.d.
**46**	n.d.	n.d.	n.d.	n.d.	n.d.	n.d.	n.d.	n.d.	n.d.	n.d.	n.d.
**47**	n.d.	n.d.	n.d.	n.d.	n.d.	n.d.	n.d.	n.d.	n.d.	< 0.19	n.d.
**48**	n.d.	n.d.	n.d.	n.d.	n.d.	n.d.	n.d.	n.d.	n.d.	3.74±2.33	n.d.
**49**	n.d.	n.d.	n.d.	n.d.	n.d.	n.d.	n.d.	n.d.	n.d.	27.6±2.11	n.d.
**50**	n.d.	> 117	n.d.	n.d.	n.d.	n.d.	n.d.	n.d.	n.d.	26.2±2.12	n.d.

AZO: azoxystrobin; **DMX**: dimoxystrobin; **IMZ**: imazalil; **FIP**: fipronil; **IPC**: ipconazole; **PEN**: penconazole; **PCL**: prochloraz; **MTZ**: metconazole; **BZT**: benzotriazole; **TRC**: tetraconazole; **OCT**: octocrylene

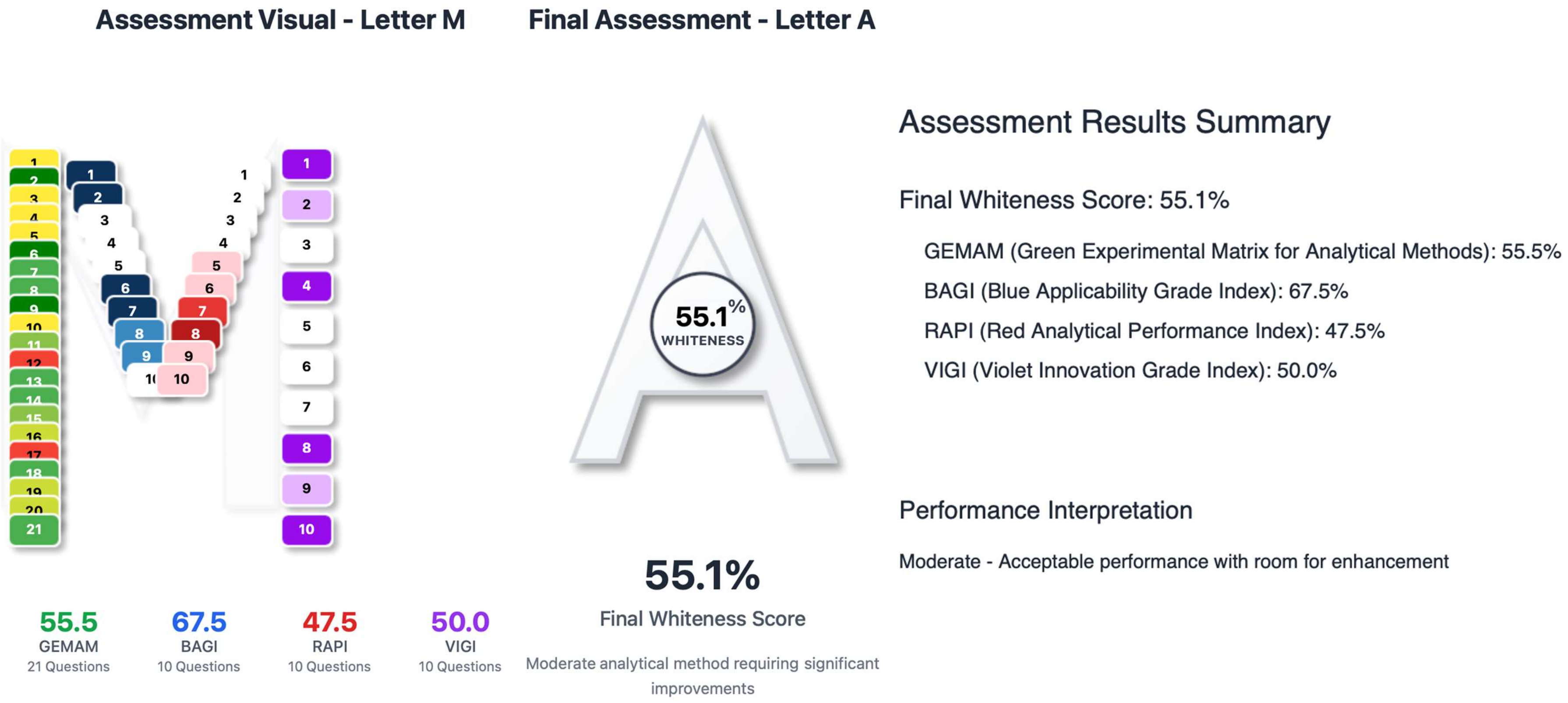

Figure S1 *MA whiteness assessment score of the SPE-LC-MS/MS method developed to determine 23 MPs in DW.*
